# Supplementary material for: iIMPACT: integrating image and molecular profiles for spatial transcriptomics analysis
Source: Genome Biol. 2024 Jun 6;25:147. doi: 10.1186/s13059-024-03289-5 (PMC11514947; doi:10.1186/s13059-024-03289-5)
Supplement: Supplementary file 1 — Additional file 1. Supplementary materials for iIMPACT, including the supplementary sections, figures, and tables. [file 13059_2024_3289_MOESM1_ESM.pdf]

Supplementary Materials for

"iIMPACT: Integrating Image and Molecular Profiles for Spatial Transcriptomics Analysis"

Xi Jiang[1,2], **Shidan Wang**[1], **Lei Guo**[1], **Bencong Zhu**[3,4], **Zhuoyu Wen**[1], **Liwei Jia**[5],
Lin Xu[1,*], **Guanghua Xiao**[1,*], and **Qiwei Li**[4,*]

[1]Quantitative Biomedical Research Center,
Peter O'Donnell Jr. School of Public Health,
The University of Texas Southwestern Medical Center,
Dallas, TX 75390, United States

[2]Department of Statistics and Data Science,
Southern Methodist University,
Dallas, TX 75275, United States

[3]Department of Statistics,
The Chinese University of Hong Kong,
Hong Kong SAR, China

[4]Department of Mathematical Sciences,
The University of Texas at Dallas,
Richardson, TX 75080, United States

[5]Department of Pathology,
The University of Texas Southwestern Medical Center,
Dallas, TX 75390, United States

S1. Evaluation of spaDEG detection using Moran's I

To quantitatively assess the spatial pattern strength of spaDEGs identified by iIMPACT and SVGs detected by alternative methods, we computed Moran's I [38], a commonly used metric for measuring spatial autocorrelation. This index varies between -1 and 1, where higher absolute values indicate a stronger spatial correlation. In particular, for each gene j of interest,

$$\text{Moran's I}(j) = \frac{N}{\sum_{i=1}^{N}\sum_{i'=1}^{n} g_{ii'}} \frac{\sum_{i=1}^{N}\sum_{i'=1}^{n} g_{ii'}(c_{ij}-\bar{c}_j)(c_{i'j}-\bar{c}_j)}{\sum_{i=1}^{N}(c_{ij}-\bar{c}_j)^2}, \tag{1}$$

where c_{ij} is the gene expression count observed on spot i and $\bar{c}_j = \sum_{i=1}^{N} c_{ij}/N$ is the average expression of gene j over all the N spots. Note that $\boldsymbol{G}$ is the neighborhood information matrix derived from the SRT geospatial profile. Fig. S5 displays the average Moran's I for spaDEGs/SVGs identified by iIMPACT, SpaGCN, SpatialDE, and SPARK across three SRT datasets, using varying selection thresholds for top genes. The boxplots comparing the top $1,000$ spaDEGs/SVGs detected by these four methods reveal that, in general, spaDEGs uncovered by iIMPACT exhibit better spatial variability and stronger spatial patterns compared to SVGs identified by the other methods.

S2. Simulation Study

Data generative model: The simulated data were generated based on the $K = 5$ histology-based spatial domains identified by iIMPACT on the human breast cancer dataset (Fig. 3B). The posterior means of those domain-specific relative abundances of cell types $\hat{\boldsymbol{\omega}}_k$'s and mean vectors of low-dimensional representation of gene expression $\hat{\boldsymbol{\mu}}_k$'s are given in Table S7. Using this real data information, we generated the cell type abundance for each spot from a multinomial distribution

$$\boldsymbol{v}_i | z_i = k \;\sim\; \text{Multi}(m_i, \hat{\boldsymbol{\omega}}_k),$$

where the size parameter m_i was also obtained from real data. For generating high-dimensional gene expression counts, we first projected the P'-dimensional domain-specific mean vectors $\hat{\boldsymbol{\mu}}_k$'s on to the original basis, denoted by a P-dimensional vector $\tilde{\boldsymbol{\mu}}_k$. To mimic the excess zeros and over-dispersion, we sampled each gene expression count c_{ij} from a zero-inflated negative binomial (ZINB) distribution,

$$c_{ij} | z_i = k \;\sim\; \pi_i \mathrm{I}(c_{ij} = 0) + (1 - \pi_i)\mathrm{NB}(s_i \exp(\tilde{\mu}_{kj}), \psi_j), \tag{2}$$

where the size factors s_i's were sampled from a log-normal distribution with mean 0 and standard deviation 0.2, i.e., $s_i \sim \mathrm{LN}(0, 0.2)$. The dispersion parameters ψ_j's were sampled from an exponential distribution, i.e., $\psi_j \sim \mathrm{Exp}(\lambda_\psi)$, with two choices of the rate parameter $\lambda_\psi = 0.1$ or 0.2, corresponding to low and high variability. The false zero proportion parameters π_i's were set to be 0.3 or 0.5, corresponding to low and high sparsity. For each of the four scenarios in terms of λ_ψ and π_i, we independently repeated the above steps to generate 10 replicated simulated datasets.

iIMPACT settings: We chose the number of reduced dimensions as $P' = 3$ in the PCA step for obtaining the low-dimensional representation of gene expression levels $\boldsymbol{Y}$. The number of histology-based spatial domains was fixed at $K = 5$. We followed the recommended

prior setting, as detailed in the METHODS section. Regarding the image profile weight, we set $w = 1$ as the simulated image and molecular profiles (i.e., $\boldsymbol{V}$ and $\boldsymbol{Y}$) were generated independently. As for the MCMC algorithm, we ran four independent MCMC chains with $U = 10,000$ iterations, discarding the first half as burn-in. We started each chain from a model by randomly drawing all parameters from their prior distributions. The results reported in Fig. S22 were obtained by pooling the MCMC outputs from the four chains after.

Competing methods: We compared the performance of iIMPACT on spatial domain identification with six current state-of-the-art methods, SpaGCN [19], BayesSpace [17], BASS [18], stLearn [20], MUSE [21], and Louvain [14]. We used the default setting of each competing method, as suggested by the authors. The number of spatial domains was fixed as $K = 5$ for all methods.

Results: We quantified the clustering performance *via* the widely used adjusted Rand index (ARI). It ranges from 0 to 1, with higher values indicating greater consistency between the clustering results and the ground truth. The results are shown in Fig. S22. iIMPACT substantially outperformed all other methods, exhibiting the highest average ARI under all four scenarios, which highlights the benefit of integrating the cell type abundance information into the spatial domain identification process. BASS achieved very high ARIs compared with other competing methods, even though it did not utilize the information from the histology image. Besides, it had the relatively small variance of ARIs among different replicates, indicating its robust performance regarding of the random noise in the SRT data. SpaGCN also demonstrated superior performance, leveraging its ability to utilize histology information effectively. Conversely, stLearn and MUSE, despite its capability to incorporate histology images, had unsatisfactory clustering accuracy and performed similarly to Louvain, a non-spatial clustering method. BayesSpace had a large variance among replicates since it

might fail to converge for some replicates. Comparing the performance under low and high variability settings, iIMPACT was robust to the level of over-dispersion of gene expression counts due to the normalization and dimensionality reduction procedures employed before the clustering model. However, it suffered from reduced clustering accuracy under high-sparsity settings.

S3. Application to human DLPFC data

We demonstrated iIMPACT's proficiency in analyzing SRT data from normal tissue sections by applying it to the LIBD human DLPFC dataset from 10x Visium platform [52]. This study measured gene expression across 12 tissue slices obtained from the DLPFC in three human brains. For our analysis, we focused on tissue slice 151510. The manual annotation of the tissue layers (see Fig. S14) provided by the original study was used to benchmark the spatial domain detection accuracy of various methods.

To assess iIMPACT's flexibility with various nuclei identification methods, we applied Hover-Net [28] to extract the cellular information from the paired histology image. Hover-Net is a state-of-the-art deep learning architecture specifically designed for segmentation and classification of nuclei in histology images. It combines three branches: the nuclear pixel branch separating nuclear pixels, the HoVer branch for spatial distance map prediction, and the nuclear classification branch identifying the type of each nucleus. Although Hover-Net was trained across multiple cancer datasets, it lacks specific training for cell type classification in normal brain tissues. Consequently, its classification capabilities are constrained for the DLPFC histology images. Therefore, we leveraged Hover-Net solely for the segmentation of nuclei to assemble the cell type abundance table $\boldsymbol{V}$. Specifically, we only use the total count of identified cell nucleis within each spot and its expanded area, represented as m_i, in this phase.

To determine the cell type composition within each spot, we employed STDeconvolve [51],

a reference-free cell type deconvolution method. STdeconvolve builds on latent Dirichlet allocation (LDA) model and does not rely the on single-cell transcriptomics references, which improves its applicability on SRT data. Applying STdeconvolve to the human DLPFC data, we identified $Q = 5$ unlabeled cell types and deconvolved their proportions per spot, represented as $(\tilde{v}_{i1}, \ldots, \tilde{v}_{iQ})^\top$. Each entry v_{iq} in the cell type abundance table was then constructed by multiplying the Hover-Net-identified nuclei count m_i with the STDeconvolve-estimated cell type proportion $\tilde{v}_{iq}$, followed by rounding to the nearest integer. This can be formulated as $v_{iq} = \lceil m_i \tilde{v}_{iq} \rfloor$.

We created the image profile following the above two steps, which are also illustrated in Fig. S13. The molecular and geospatial profiles were generated following the procedure shown in Fig. 1. After assembling these three profiles, we implemented iIMPACT and compared its spatial domain identification performance with five other methods: SpaGCN, BayesSpace, BASS, stLearn, and MUSE. As shown in Fig. S14, iIMPACT achieved the highest ARI of 0.507, indicating a strong concordance with the manually annotated tissue layers. These findings underscore the viability of the alternative data preparation approach for iIMPACT, accommodating user preferences for specific nuclei segmentation and classification techniques. Moreover, the results attest to iIMPACT's versatility in handling various tissue types in SRT data analysis.

S4. Details of the MCMC Algorithm

According to the model description in the METHODS section, the full data likelihood of the proposed Bayesian finite normal-multinomial mixture model is given as follows.

$$
\begin{aligned}
&f(\boldsymbol{Y}, \boldsymbol{V}|\boldsymbol{z}, \boldsymbol{\mu}_1, \ldots, \boldsymbol{\mu}_K, \boldsymbol{\Sigma}_1, \ldots, \boldsymbol{\Sigma}_K, \boldsymbol{\omega}_1, \ldots, \boldsymbol{\omega}_K) \\
=&\prod_{k=1}^{K}\prod_{i=1}^{N} \mathrm{I}(z_i = k) f(\boldsymbol{y}_i, \boldsymbol{v}_i | z_i = k, \boldsymbol{\mu}_k, \boldsymbol{\Sigma}_k, \boldsymbol{\omega}_k),
\end{aligned}
$$

where $\mathrm{I}(\cdot)$ denotes the indicator function and

$$
\begin{aligned}
&f(\boldsymbol{y}_i, \boldsymbol{v}_i | z_i = k, \boldsymbol{\mu}_k, \boldsymbol{\Sigma}_k, \boldsymbol{\omega}_k) \\
&= f(\boldsymbol{y}_i | z_i = k, \boldsymbol{\mu}_k, \boldsymbol{\Sigma}_k) f(\boldsymbol{v}_i | z_i = k, \boldsymbol{\omega}_k)^w \\
&= \mathrm{MN}(\boldsymbol{y}_i; \boldsymbol{\mu}_k, \boldsymbol{\Sigma}_k) \mathrm{Multi}(\boldsymbol{v}_i; m_i, \boldsymbol{\omega}_k)^w \\
&= (2\pi)^{-P'/2} |\boldsymbol{\Sigma}_k|^{-1/2} \exp\left(-\frac{1}{2}(\boldsymbol{y}_i - \boldsymbol{\mu}_k)^\top \boldsymbol{\Sigma}_k^{-1} (\boldsymbol{y}_i - \boldsymbol{\mu}_k)\right) \left(\frac{m_i!}{\prod_{q=1}^{Q} v_{iq}!} \prod_{q=1}^{Q} \omega_{kq}^{v_{iq}}\right)^w \\
&\propto |\boldsymbol{\Sigma}_k|^{-1/2} \exp\left(-\frac{1}{2}(\boldsymbol{y}_i - \boldsymbol{\mu}_k)^\top \boldsymbol{\Sigma}_k^{-1} (\boldsymbol{y}_i - \boldsymbol{\mu}_k)\right) \left(\prod_{q=1}^{Q} \omega_{kq}^{v_{iq}}\right)^w .
\end{aligned}
$$

We assume an independent prior structure 1) between the normal and multinomial sub-components; and 2) among their parameters belonging to different spatial domains. Thus, the joint distribution of priors for parameters can be written as,

$$
\begin{aligned}
&\pi(\boldsymbol{z}, \boldsymbol{\mu}_1, \ldots, \boldsymbol{\mu}_K, \boldsymbol{\Sigma}_1, \ldots, \boldsymbol{\Sigma}_K, \boldsymbol{\omega}_1, \ldots, \boldsymbol{\omega}_K) \\
&= \pi(\boldsymbol{z}) \pi(\boldsymbol{\mu}_1, \ldots, \boldsymbol{\mu}_K, \boldsymbol{\Sigma}_1, \ldots, \boldsymbol{\Sigma}_K) \pi(\boldsymbol{\omega}_1, \ldots, \boldsymbol{\omega}_K)^w \\
&= \pi(\boldsymbol{z}) \prod_{k=1}^{K} \pi(\boldsymbol{\mu}_k, \boldsymbol{\Sigma}_k) \prod_{k=1}^{K} \pi(\boldsymbol{\omega}_k)^w \\
&= \pi(\boldsymbol{z}) \prod_{k=1}^{K} \pi(\boldsymbol{\mu}_k | \boldsymbol{\Sigma}_k) \pi(\boldsymbol{\Sigma}_k) \prod_{k=1}^{K} \pi(\boldsymbol{\omega}_k)^w.
\end{aligned}
$$

We assign the Markov random field (MRF) prior for histology-based spatial domain indicator

$\boldsymbol{z}$ as

$$\pi(z_i|\boldsymbol{z}_{-i}) \propto \exp\left(d_k + f \sum_{i'=1, i'\neq i}^{N} g_{ii'} \mathrm{I}(z_{i'} = k)\right),$$

We assign the conjugate priors for other parameters, listed as follows, so that the Gibbs sampler can be applied for posterior sampling.

$$\boldsymbol{\mu}_k|\boldsymbol{\Sigma}_k \sim \mathrm{MN}(\boldsymbol{\nu}_0, \boldsymbol{\Sigma}/\tau_0)$$

$$\text{or equivalently, } \pi(\boldsymbol{\mu}_k|\boldsymbol{\Sigma}_k) = (2\pi/\tau_0)^{-P'/2}|\boldsymbol{\Phi}_0|^{-1/2} \exp\left(-\frac{\tau_0}{2}(\boldsymbol{\mu}_k - \boldsymbol{\nu}_0)^\top \boldsymbol{\Phi}_0^{-1}(\boldsymbol{\mu}_k - \boldsymbol{\nu}_0)\right),$$

$$\boldsymbol{\Sigma}_k \sim \mathrm{IW}(\eta_0, \boldsymbol{\Phi}_0)$$

$$\text{or equivalently, } \pi(\boldsymbol{\Sigma}_k) = \frac{|\boldsymbol{\Phi}_0|^{\eta_0/2}}{2^{\eta_0 P'/2}\Gamma_{P'}(\eta_0/2)}|\boldsymbol{\Sigma}_k|^{-(\eta_0+P'+1)/2} \exp\left(-\frac{1}{2}\mathrm{tr}\left(\boldsymbol{\Phi}_0\boldsymbol{\Sigma}_k^{-1}\right)\right),$$

and

$$\boldsymbol{\omega}_k \sim \mathrm{Dir}(\boldsymbol{\alpha}_0) \text{ or equivalently, } \pi(\boldsymbol{\omega}_k) = \frac{\Gamma\left(\sum_{q=1}^{Q} \alpha_{0q}\right)}{\prod_{q=1}^{Q} \Gamma(\alpha_{0q})} \prod_{q=1}^{Q} \omega_{kq}^{\alpha_{0q}-1},$$

where $\Gamma_{P'}(\cdot)$ and $\Gamma(\cdot)$ denote the P'-dimensional and univariate gamma function.

We recommend a weakly informative prior setting by choosing the MRF hyperparameters $d_1 = \ldots = d_K = 1$ and $f = 1$, the multivariate normal hyperparameters $\boldsymbol{\nu}_0 = \frac{1}{N}\sum_{i=1}^{N} \boldsymbol{y}_i$, $\tau_0 = 0.01$, $\eta_0 = P' + 1$, and $\boldsymbol{\Phi}_0 = \boldsymbol{I}_{P'\times P'}$ (i.e., the P'-by-P' identity matrix), and the multinomial hyperparameters $\alpha_{01} = \ldots = \alpha_{0Q} = 1$.

The full posterior distribution of the proposed Bayesian normal-multinomial mixture model is given in the following formula.

$$\pi(\boldsymbol{z}, \boldsymbol{\mu}_1, \ldots, \boldsymbol{\mu}_K, \boldsymbol{\Sigma}_1, \ldots, \boldsymbol{\Sigma}_K, \boldsymbol{\omega}_1, \ldots, \boldsymbol{\omega}_K|\boldsymbol{Y}, \boldsymbol{V}) \propto$$

$$f(\boldsymbol{Y}, \boldsymbol{V}|\boldsymbol{z}, \boldsymbol{\mu}_1, \ldots, \boldsymbol{\mu}_K, \boldsymbol{\Sigma}_1, \ldots, \boldsymbol{\Sigma}_K, \boldsymbol{\omega}_1, \ldots, \boldsymbol{\omega}_K)\pi(\boldsymbol{z}, \boldsymbol{\mu}_1, \ldots, \boldsymbol{\mu}_K, \boldsymbol{\Sigma}_1, \ldots, \boldsymbol{\Sigma}_K, \boldsymbol{\omega}_1, \ldots, \boldsymbol{\omega}_K)$$

Posterior sampling is employed by the MCMC algorithm. Our primary interest lies in identifying histology-based spatial domains and the interactive zone *via* inferring the spatial domain indicator vector $\boldsymbol{z}$, and in characterizing domain-specific relative abundance of cell types *via* inferring $\boldsymbol{\omega}_1, \ldots, \boldsymbol{\omega}_K$. Since we use conjugate priors on all model parameters,

$\boldsymbol{z}, \boldsymbol{\mu}_1, \ldots, \boldsymbol{\mu}_K, \boldsymbol{\Sigma}_1, \ldots, \boldsymbol{\Sigma}_K, \boldsymbol{\omega}_1, \ldots, \boldsymbol{\omega}_K$, their conditional distributions are all in closed form and easy to sample from. Consequently, we can rely on the Gibbs sampler, an MCMC algorithm for obtaining a sequence of observations approximated from a multivariate probability distribution when direct sampling is difficult. To be specific, we perform the following steps sequentially at each MCMC iteration after a random initialization.

Update the histology-based spatial domain indicator $\boldsymbol{z}$: We update $z_1, \ldots, z_N$ sequentially. To allocate spot i to one of the K histology-based spatial domains, we sample z_i from a single-drawing multinomial distribution,

$$z_i|\cdot \sim \text{Multi}(1, (\pi(z_i = 1|\cdot)/e, \ldots, \pi(z_i = K|\cdot)/e)),$$

where

$$\begin{aligned}\pi(z_i = k|\cdot) &\propto f(\boldsymbol{y}_i, \boldsymbol{v}_i | z_i = k, \boldsymbol{\mu}_k, \boldsymbol{\Sigma}_k, \boldsymbol{\omega}_k)\pi(z_i = k|\boldsymbol{z}_{-i}) \\ &\propto |\boldsymbol{\Sigma}_k|^{-1/2} \exp\left(-\frac{1}{2}(\boldsymbol{y}_i - \boldsymbol{\mu}_k)^\top \boldsymbol{\Sigma}_k^{-1}(\boldsymbol{y}_i - \boldsymbol{\mu}_k)\right)\left(\prod_{q=1}^{Q} \omega_{k,q}^{v_{iq}}\right)^w \\ &\quad \exp\left(d_k + f\sum_{i'=1, i'\neq i}^{N} g_{ii'}\mathrm{I}(z_{i'} = k)\right)\end{aligned}$$

and the normalization constant $e = \sum_{k=1}^{K} \pi(z_i = k|\cdot)$.

Update the domain-specific relative abundance of cell types $\boldsymbol{\omega}_k$'s: We update $\boldsymbol{\omega}_1, \ldots, \boldsymbol{\omega}_K$ sequentially. For each histology-based spatial domain k, we draw a sample of $\boldsymbol{\omega}_k$ from a Dirichlet distribution,

$$\boldsymbol{\omega}_k|\cdot \sim \text{Dir}(\boldsymbol{\alpha}_k),$$

where the concentration parameters $\boldsymbol{\alpha}_k = (\alpha_{k1}, \ldots, \alpha_{kQ})$ with each entry $\alpha_{kq} = \alpha_{0q} + \sum_{i=1}^{N} \mathrm{I}(z_i = k)v_{iq}$. Note that the last term $\sum_{i=1}^{N} \mathrm{I}(z_i = k)v_{iq}$ denotes the total number of cells with type q observed in histology-based spatial domain k.

Update the domain-specific low-dimensional representation of gene expression mean $\boldsymbol{\mu}_k$'s: We update $\boldsymbol{\mu}_1, \ldots, \boldsymbol{\mu}_K$ sequentially. For each histology-based spatial domain k,

we draw a sample of $\boldsymbol{\mu}_k$ from a multivariate normal distribution,

$$\boldsymbol{\mu}_k|\cdot \sim \mathrm{MN}(\boldsymbol{\nu}_k, \boldsymbol{\Sigma}_k/\tau_k),$$

where $\boldsymbol{\nu}_k = (\boldsymbol{\nu}_0\tau_0 + n_k\bar{\boldsymbol{y}}_k)/(\tau_0 + n_k)$ and $\tau_k = \tau_0 + n_k$. Note that $n_k = \sum_i^N \mathrm{I}(z_i = k)$ is the number of spots allocated to histology-based spatial domain k and $\bar{\boldsymbol{y}}_k = \frac{1}{n_k}\sum_i^N \mathrm{I}(z_i = k)\boldsymbol{y}_i$ denotes the average low-dimensional gene expression value over all the spots allocated to histology-based spatial domain k.

If PCA is chosen to reduce the dimension of the SRT molecular profile, then we can further set $\boldsymbol{\Sigma}_k$ to an P'-by-P' diagonal matrix due to orthogonality among principal components. In this special case, we can draw each entry in $\boldsymbol{\mu}_k$ independently,

$$\mu_{kj}|\cdot \sim \mathrm{N}(\nu_{kj}, \sigma_{kj}^2/\tau_k),$$

where $\nu_{kj} = (\nu_{0j}\tau_0 + n_k\bar{y}_{kj})/(\tau_0 + n_k)$.

Update the domain-specific covariance matrix of the low-dimensional representation of gene expression $\boldsymbol{\Sigma}_k$'s: We update $\boldsymbol{\Sigma}_1, \ldots, \boldsymbol{\Sigma}_K$ sequentially. For each histology-based spatial domain k, we draw a sample of $\boldsymbol{\Sigma}_k$ from an inverse-Wishart distribution,

$$\boldsymbol{\Sigma}_k|\cdot \sim \mathrm{IW}(\eta_k, \boldsymbol{\Phi}_k),$$

where $\eta_k = \eta_0 + n_k$ and $\boldsymbol{\Phi}_k = \boldsymbol{\Phi}_0 + \sum_{i=1}^N \mathrm{I}(z_i = k)(\boldsymbol{y}_i - \bar{\boldsymbol{y}}_k)(\boldsymbol{y}_i - \bar{\boldsymbol{y}}_k)^\top + \frac{\tau_0 n_k}{\tau_0 + n_k}(\bar{\boldsymbol{y}}_k - \boldsymbol{\nu}_0)(\bar{\boldsymbol{y}}_k - \boldsymbol{\nu}_0)^\top$.

For the case when using PCA, the inverse-Wishart prior reduces to an inverse-gamma prior, $\sigma_{kj}^2 \sim \mathrm{IG}(\eta_0/2, \phi_0/2)$. Thus, we only need to draw each diagonal entry in $\boldsymbol{\Sigma}_k$ independently,

$$\sigma_{kj}^2|\cdot \sim \mathrm{IG}(\eta_k/2, \phi_k/2),$$

where $\phi_k = \phi_0 + \sum_{i=1}^N \mathrm{I}(z_i = k)(y_{ij} - \bar{y}_{kj})^2 + \frac{\tau_0 n_k}{\tau_0 + n_k}(\bar{y}_{kj} - \nu_{0j})^2$.

S5. Sensitivity Analysis

We first conducted a sensitivity analysis to investigate how the number of dimensions reduced affects iIMPACT's performance in histology-based spatial domain identification. In particular, we varied the number of top principal components (PC) P' kept in the molecular profile from 2 to 15 and computed the ARI between the ground truth and the MAP estimate of the histology-based spatial domain indicator $\hat{\boldsymbol{z}}$. Take human breast cancer data as example, Fig. S19 summarizes the achieved ARI against different choices of P', along with the cumulative variance explained by the PCs, indicating that iIMPACT was relatively robust to the choice of number of PCs and the best performance occured when $P' = 3$.

Then, another sensitivity analysis was performed to investigate the sensitivity of iIMPACT to the choice of the image profile weight w, which controls the image profile's contribution to the spatial domain identification result. In particular, we varies w in

$$\{0.01, 0.02, 0.05, 0.10, 0.25, 0.50, 0.75, 1.00\}$$

and computed the ARI between the ground truth and the MAP estimate of the histology-based spatial domain indicator $\hat{\boldsymbol{z}}$. Fig. S21 summarizes the achieved ARI against different values of w for both the human breast cancer and mouse visual cortex STARmap data. It is interesting to know that iIMPACT was very sensitive to the choice of w. The best performance occured when setting $w = 0.05$ for the human breast cancer data from NGS-based SRT platforms (e.g., 10x Visium) and $w = 0.5$ for the mouse visual cortex data from imaging-based SRT techniques (e.g., STARmap). Therefore, to guard against under or over-fitting, the value of w should be chosen with some degree of caution.

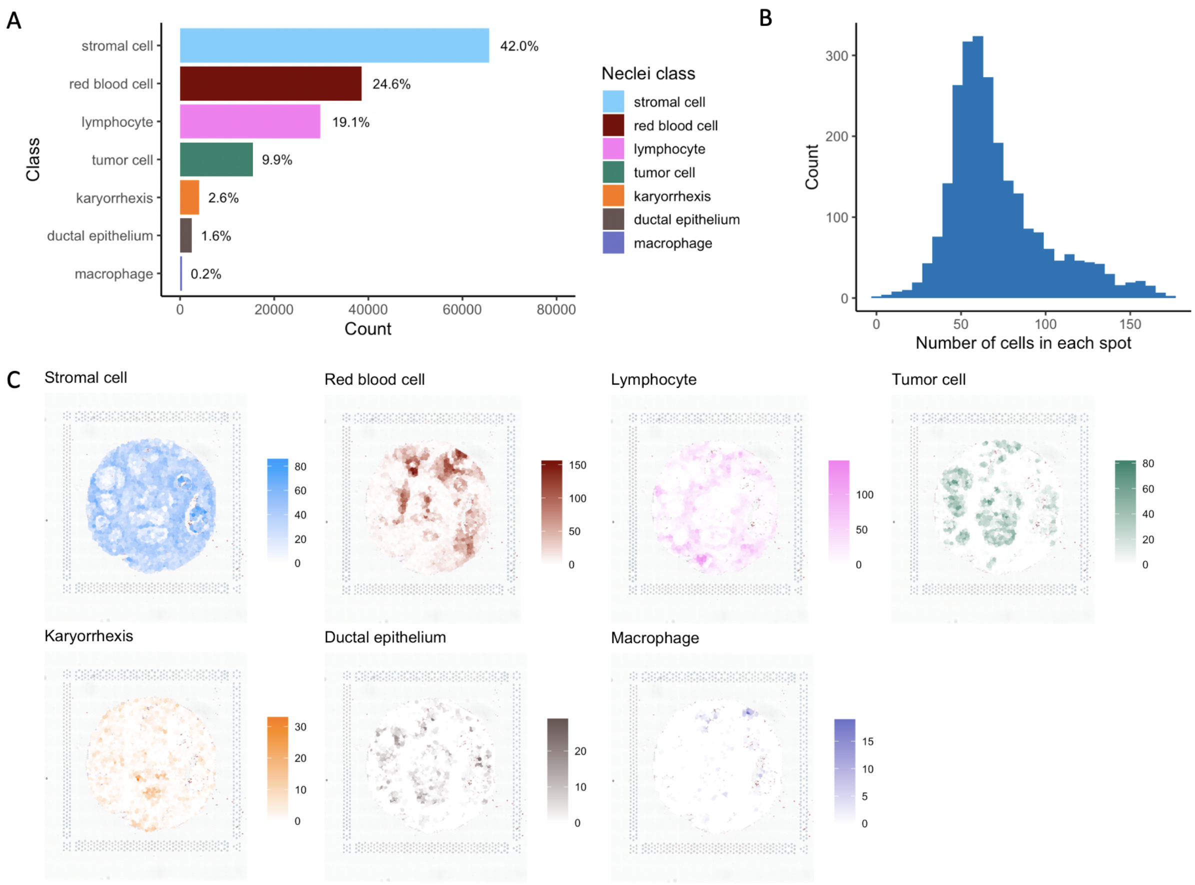

Fig. S1 Human breast cancer data: Nuclei identification results from the HD-Staining model. A. The number of nuclei identified for seven different nuclei classes; B. Histogram of number of cells in each spot expanded area; C. Spatial distribution of spot-level cell type abundance for seven nuclei classes.

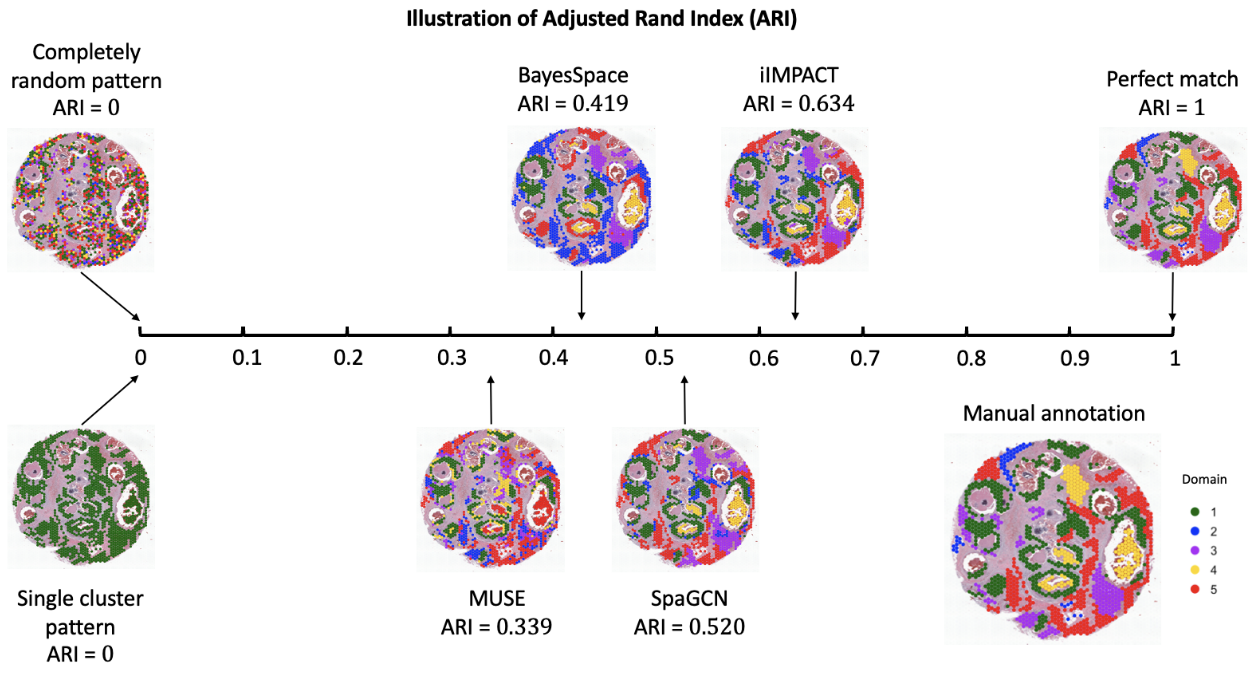

Fig. S2 A graphical illustration of the adjusted Rand index (ARI).

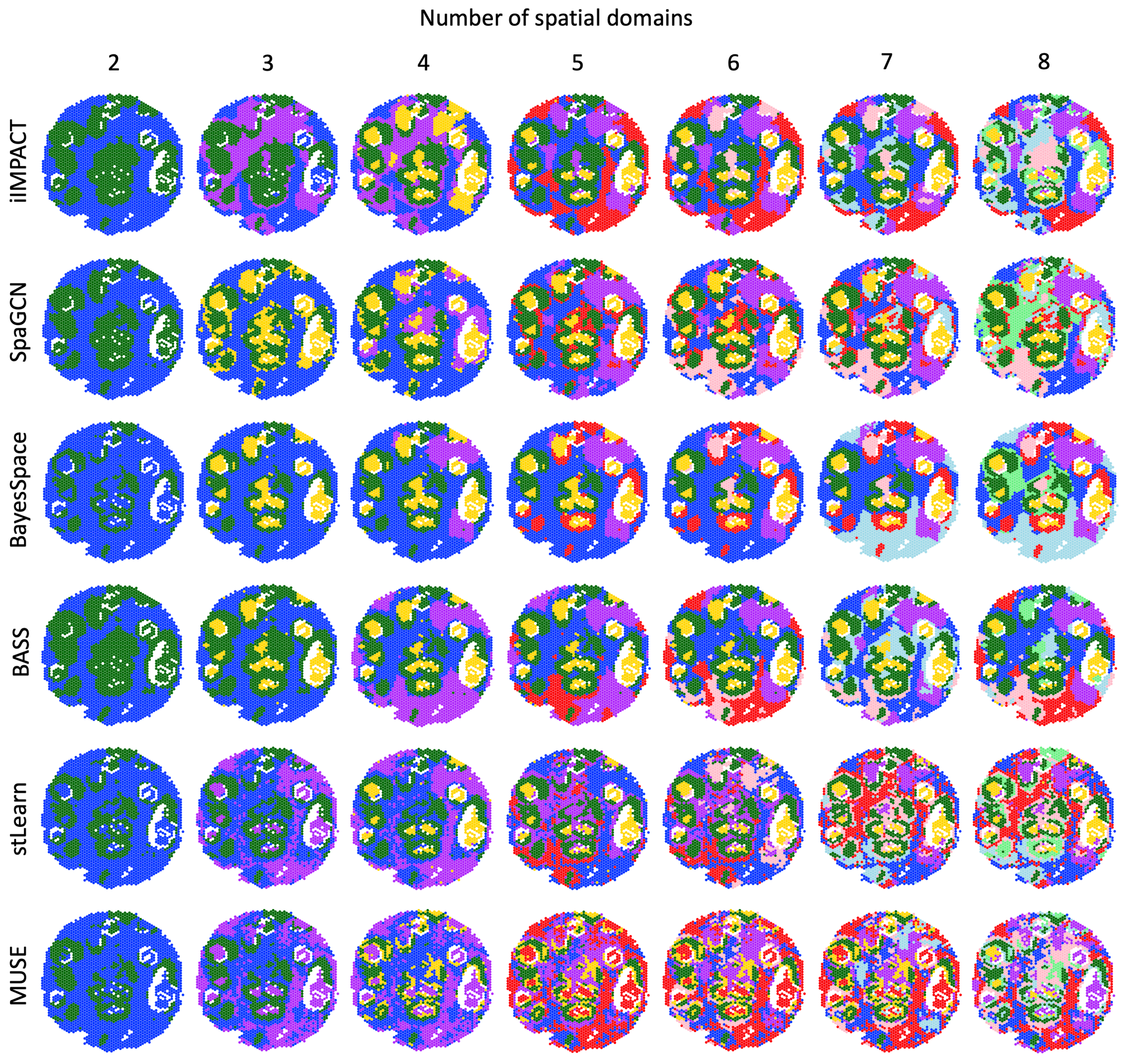

Fig. S3 Human breast cancer data: The histology-based spatial domains detected by iIMPACT, SpaGCN, BayesSpace, BASS, stLearn, and MUSE, setting the number of spatial domains K from 2 to 8.

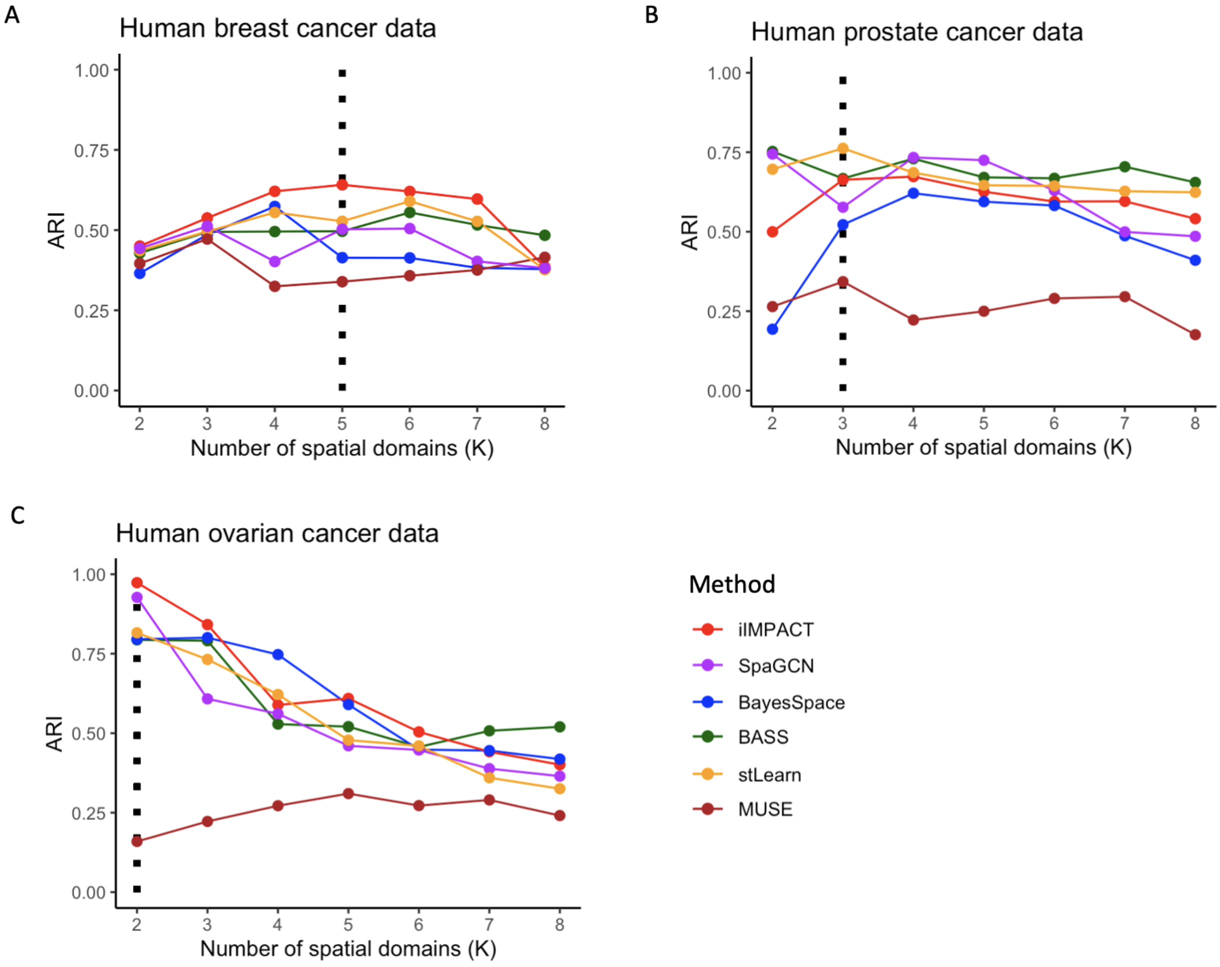

Fig. S4 ARIs achieved by six spatial domain identification methods (iIMPACT, SpaGCN, BayesSpace, BASS, stLearn, and MUSE) under different choices of the number of spatial domains K on A. human breast cancer data; B. human prostate cancer data; and C. human ovarian cancer data.

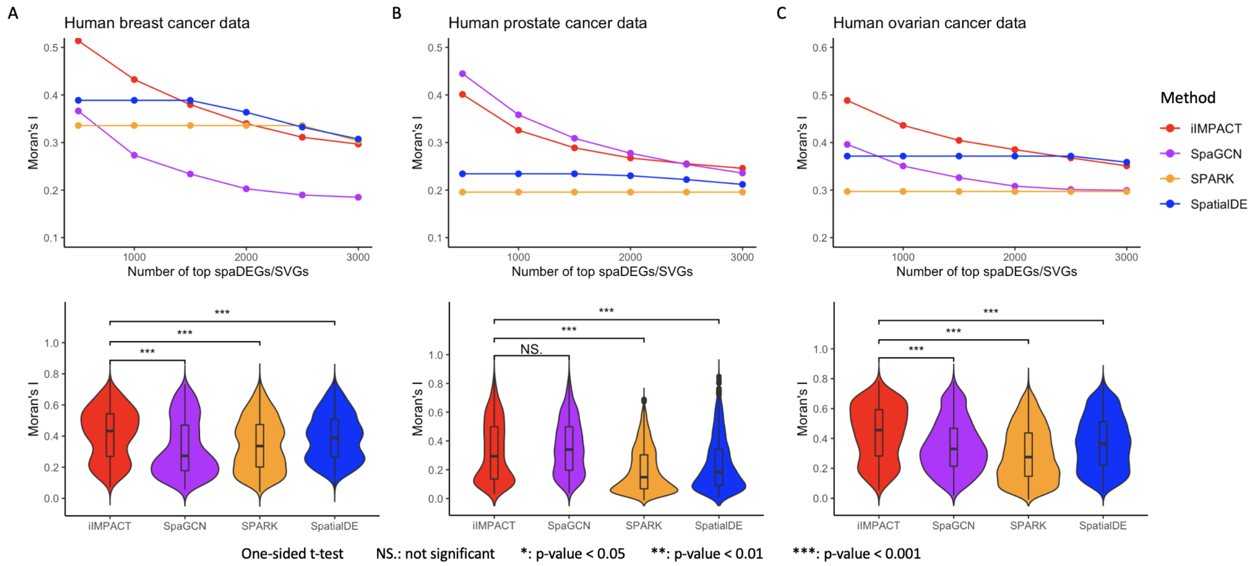

Fig. S5 Evaluation of spaDEGs detected by iIMPACT: The average Moran's I's for the spaDEGs/SVGs detected by four methods (i.e., iIMPACT, SpaGCN, SpatialDE, and SPARK) under the setting of six selection thresholds of top genes, and the boxplots of Moran's I's of top 1000 spaDEGs/SVGs detected by four methods, for the A. human breast cancer data; B. human prostate cancer data; and C. human ovarian cancer data.

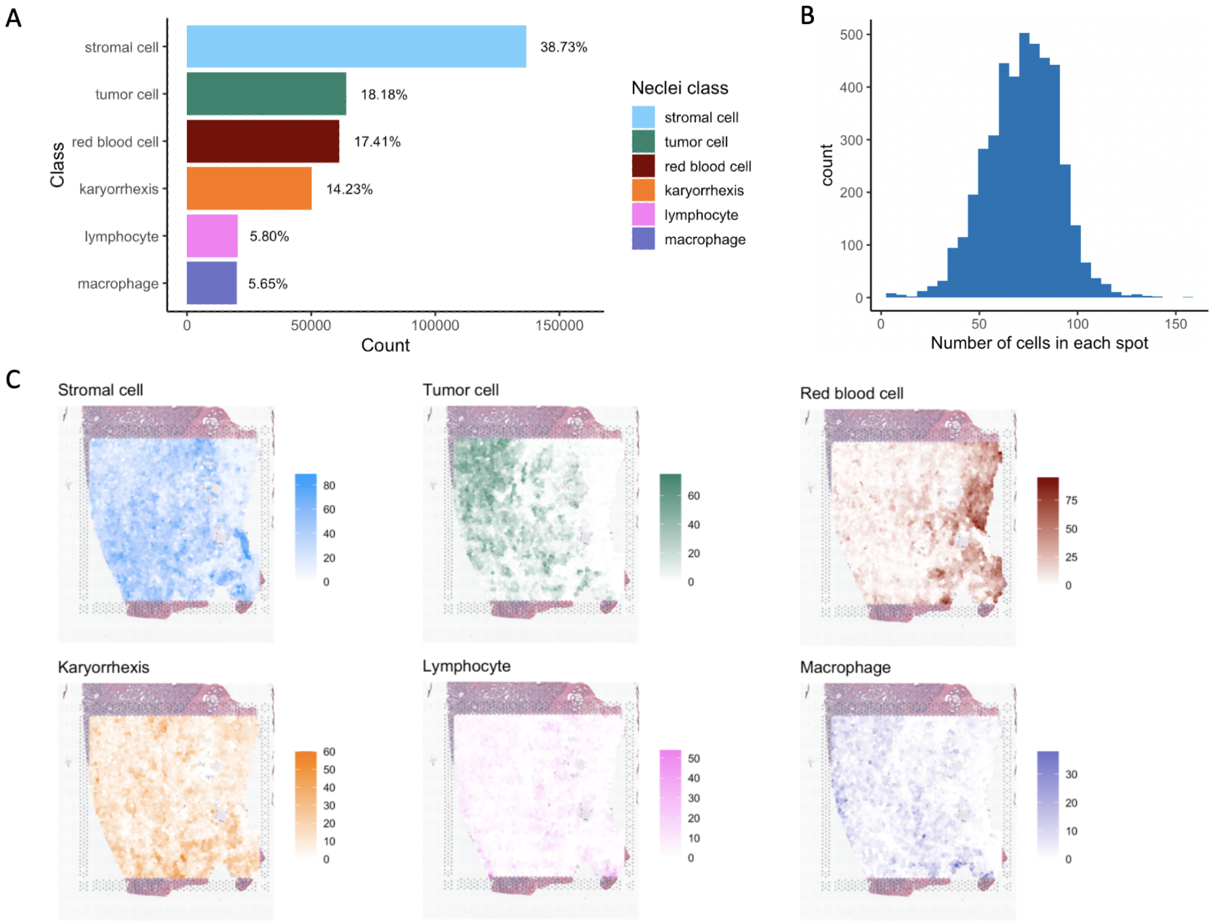

Fig. S6 Human prostate cancer data: Nuclei identification results from the HD-Staining model. A. The number of nuclei identified for six different nuclei classes; B. Histogram of number of cells in each spot expanded area; C. Spatial distribution of spot-level cell type abundance for six nuclei classes.

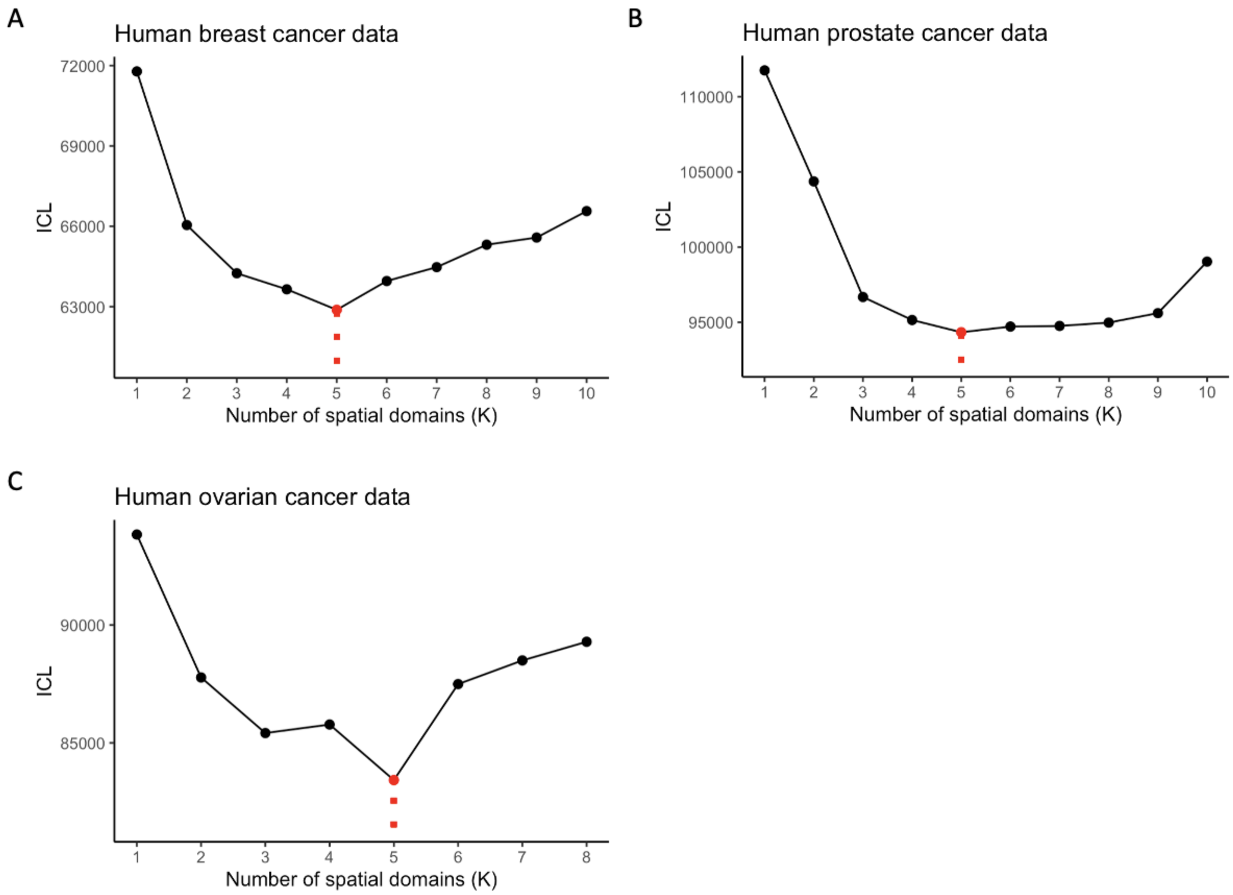

Fig. S7 Integrated completed log-likelihood (ICL) plots for the selection of the number of spatial domains K on A. human breast cancer data; B. human prostate cancer data; and C. human ovarian cancer data. The minimum at $K = 5$ was selected as the number of spatial domains to analyze for these three SRT data.

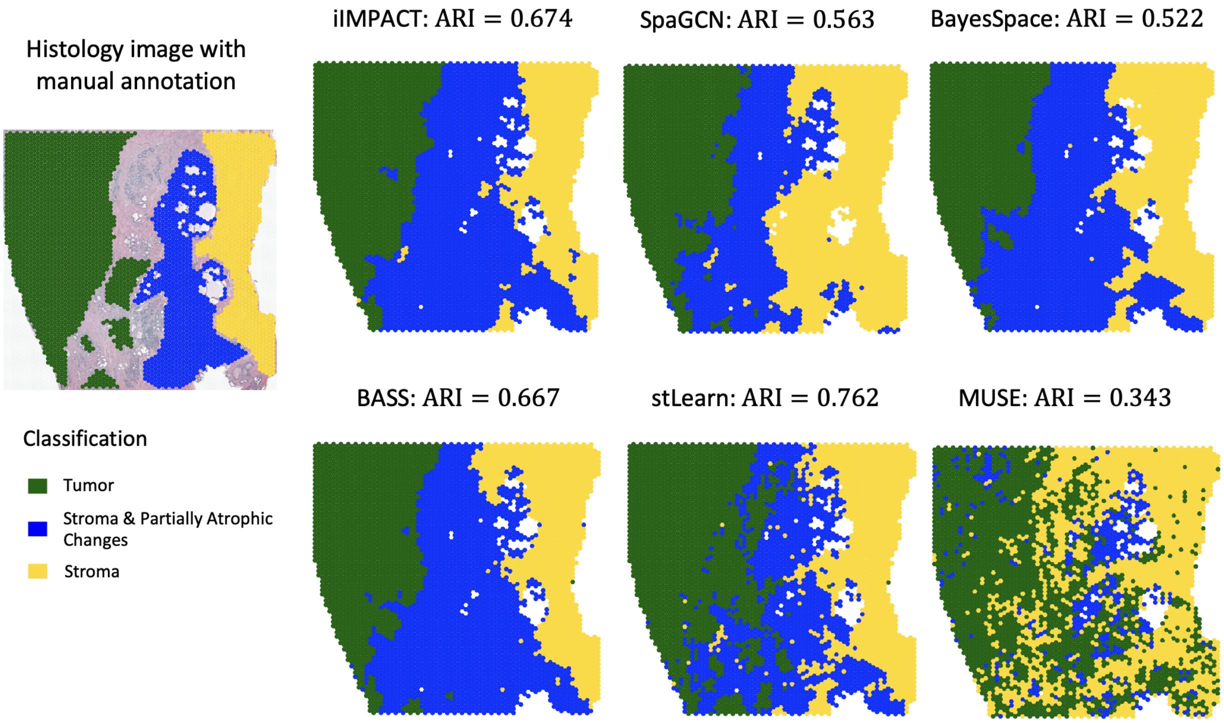

Fig. S8 Human prostate cancer data: The matching histology image of the tissue section with spot-level manually annotated labels from pathologists, and spatial domains detected by iIMPACT, SpaGCN, BayesSpace, BASS, stLearn, and MUSE, setting the number of spatial domains to be $K = 3$.

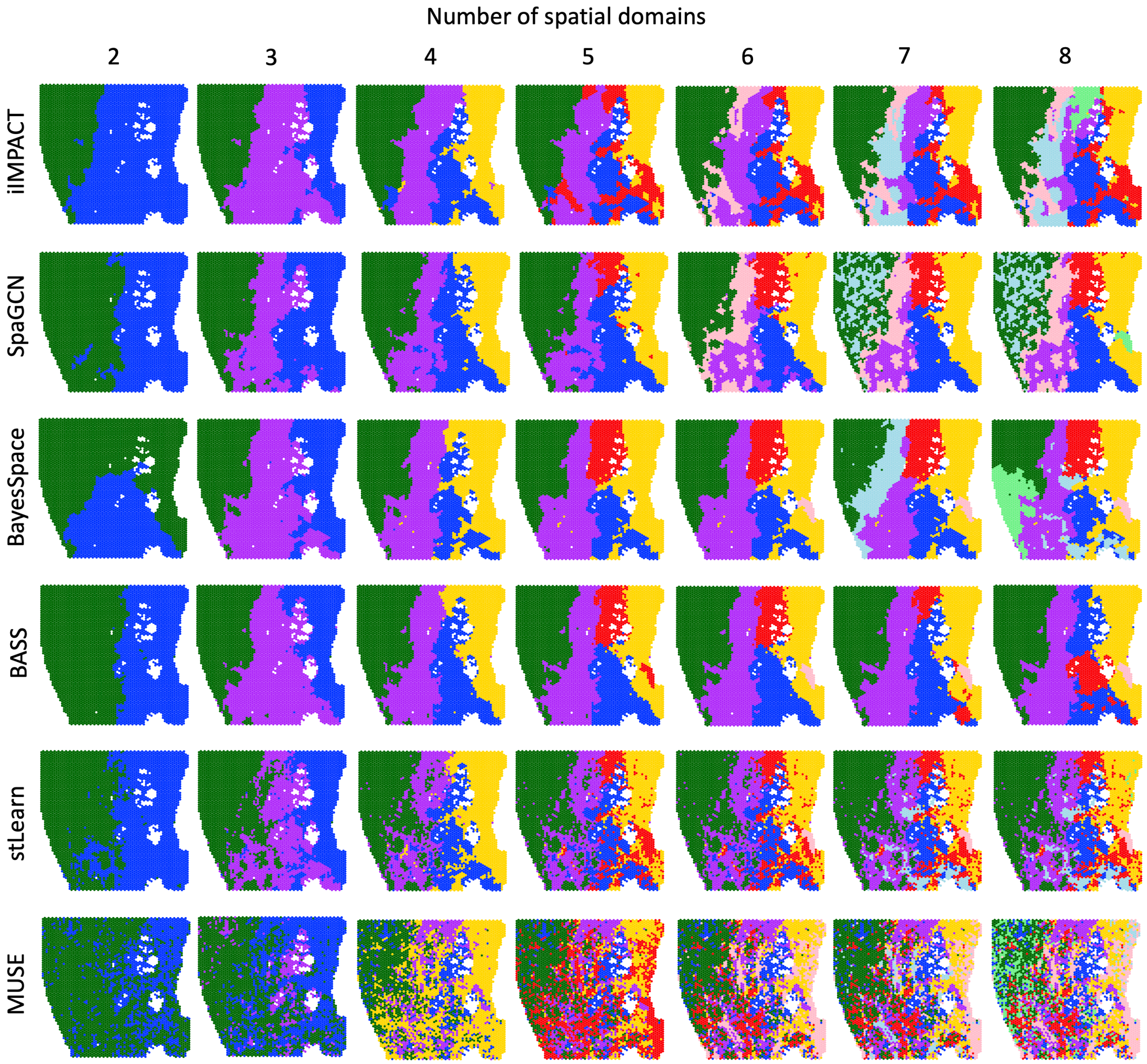

Fig. S9 Human prostate cancer data: The histology-based spatial domains detected by iIMPACT, SpaGCN, BayesSpace, BASS, stLearn, and MUSE, setting the number of spatial domains K from 2 to 8.

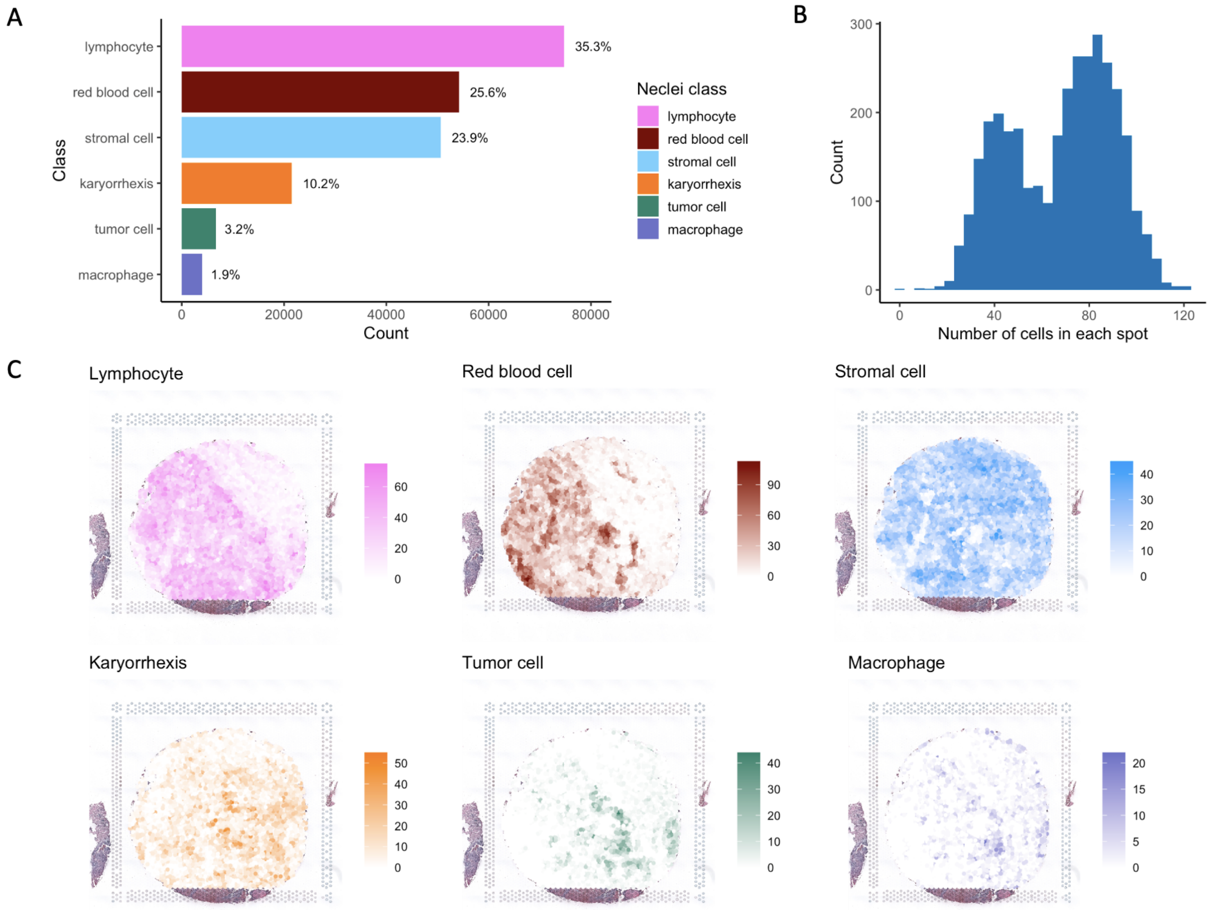

Fig. S10 Human ovarian cancer data: Nuclei identification results from the HD-Staining model. A. The number of nuclei identified for six different nuclei classes; B. Histogram of number of cells in each spot expanded area; C. Spatial distribution of spot-level cell type abundance for six nuclei classes.

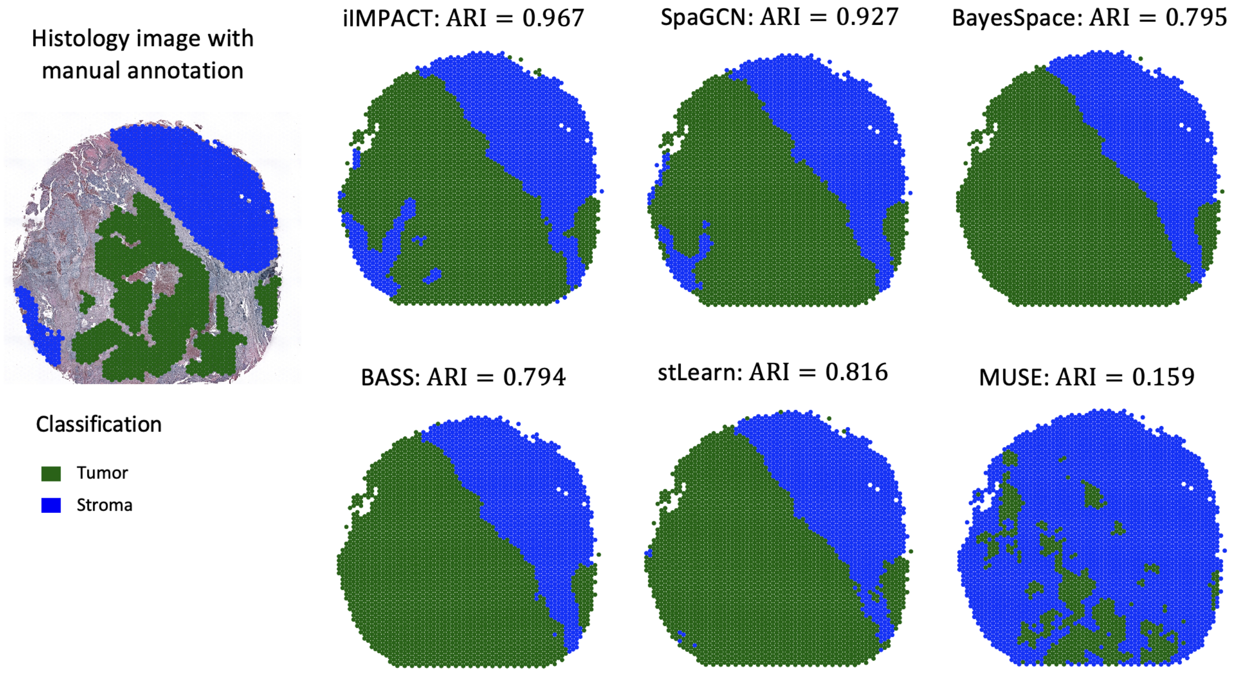

Fig. S11 Human ovarian cancer data: The matching histology image of the tissue section with spot-level manually annotated labels from pathologists, and histology-based spatial domains detected by iIMPACT, SpaGCN, BayesSpace, BASS, stLearn, and MUSE, setting the number of spatial domains to be $K = 2$.

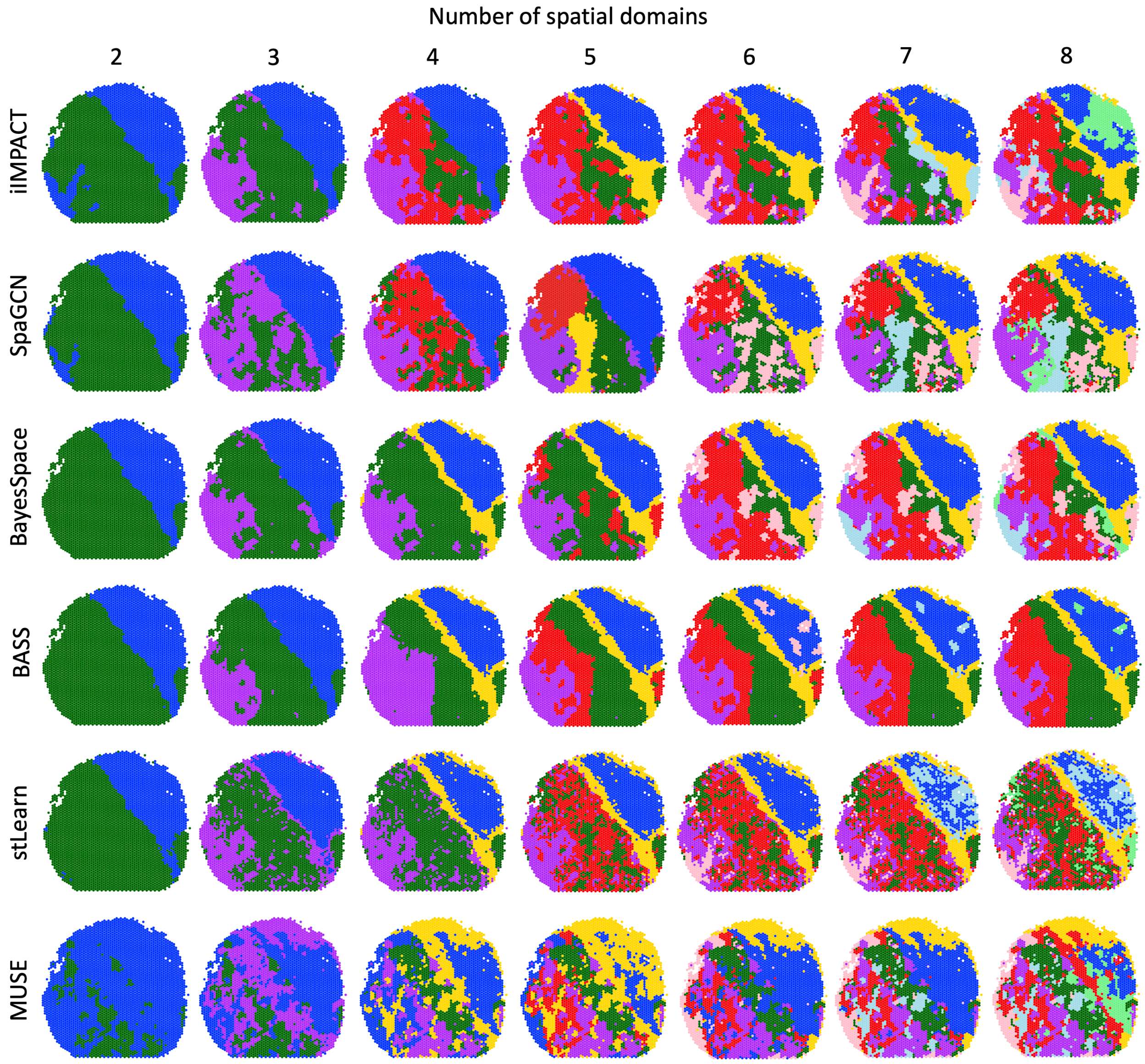

Fig. S12 Human ovarian cancer data: The histology-based spatial domains detected by iIMPACT, SpaGCN, BayesSpace, BASS, stLearn, and MUSE, setting the number of spatial domains K from 2 to 8.

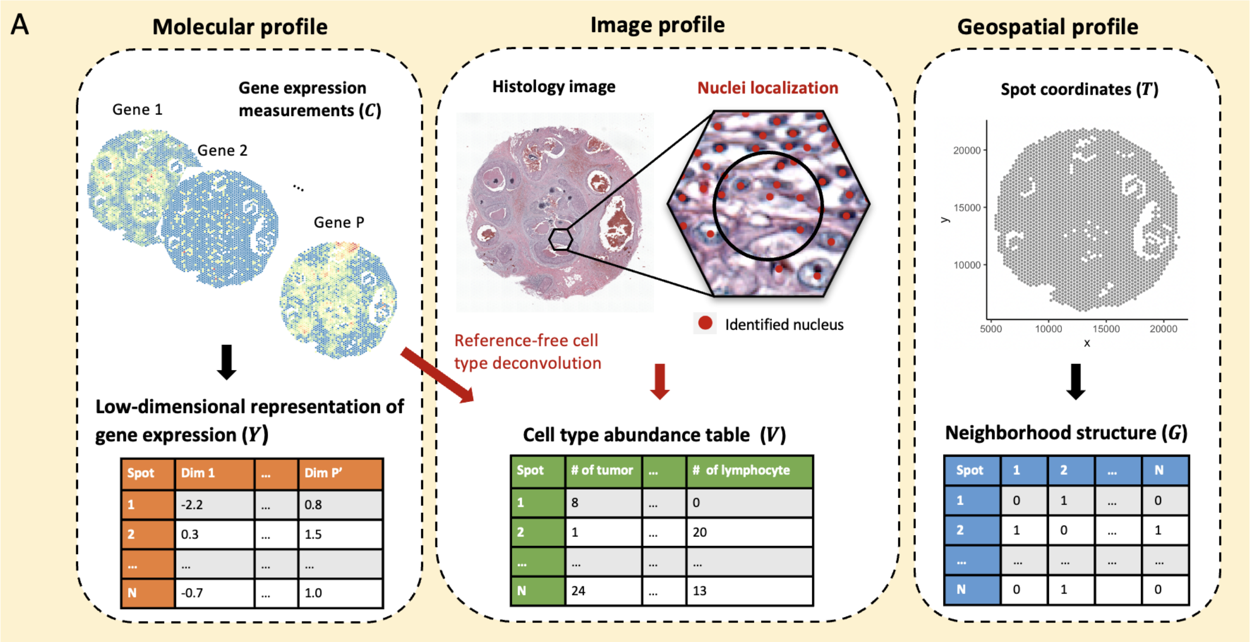

Fig. S13 An alternative pipeline for data preparation: Reference-free cell type deconvolution algorithms are applied on the molecular profile to generate the cell type abundance table, together with the nuclei localization *via* nuclei identification methods.

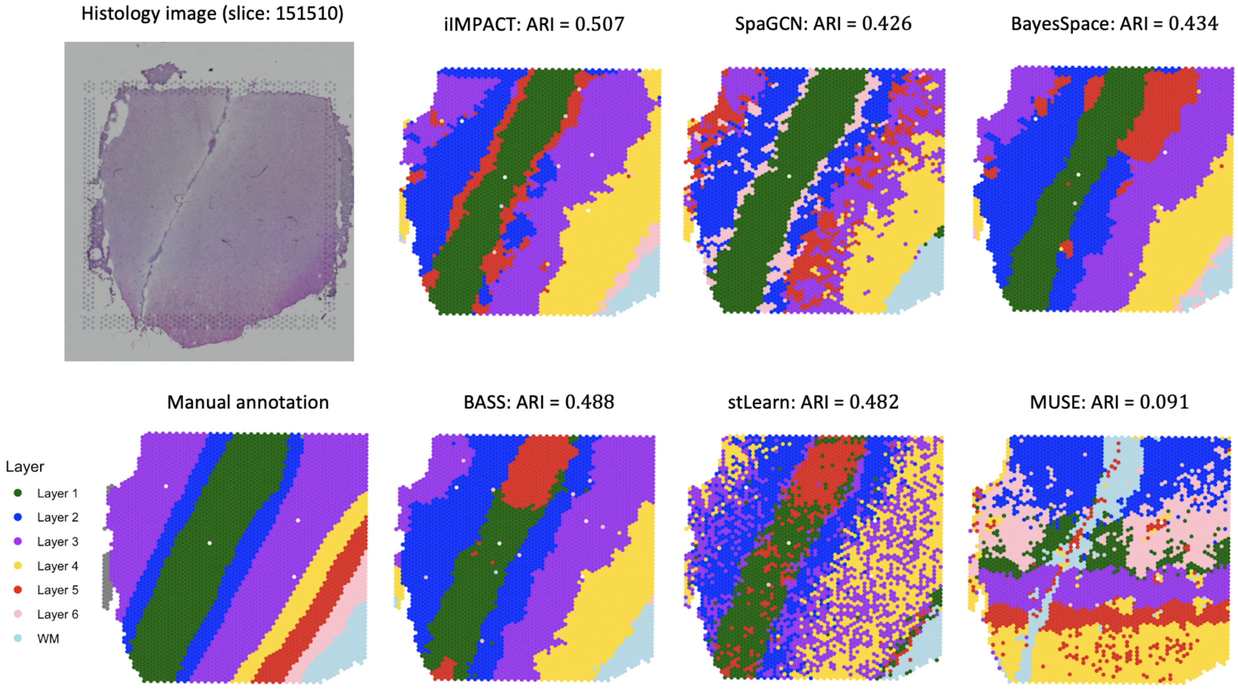

Fig. S14 LIBD human dorsolateral prefrontal cortex (DLPFC) data: The histology image of the tissue section, the manually annotation provided from the original study, and histology-based spatial domains detected by iIMPACT, SpaGCN, BayesSpace, BASS, stLearn, and MUSE, setting the number of spatial domains to be $K = 7$.

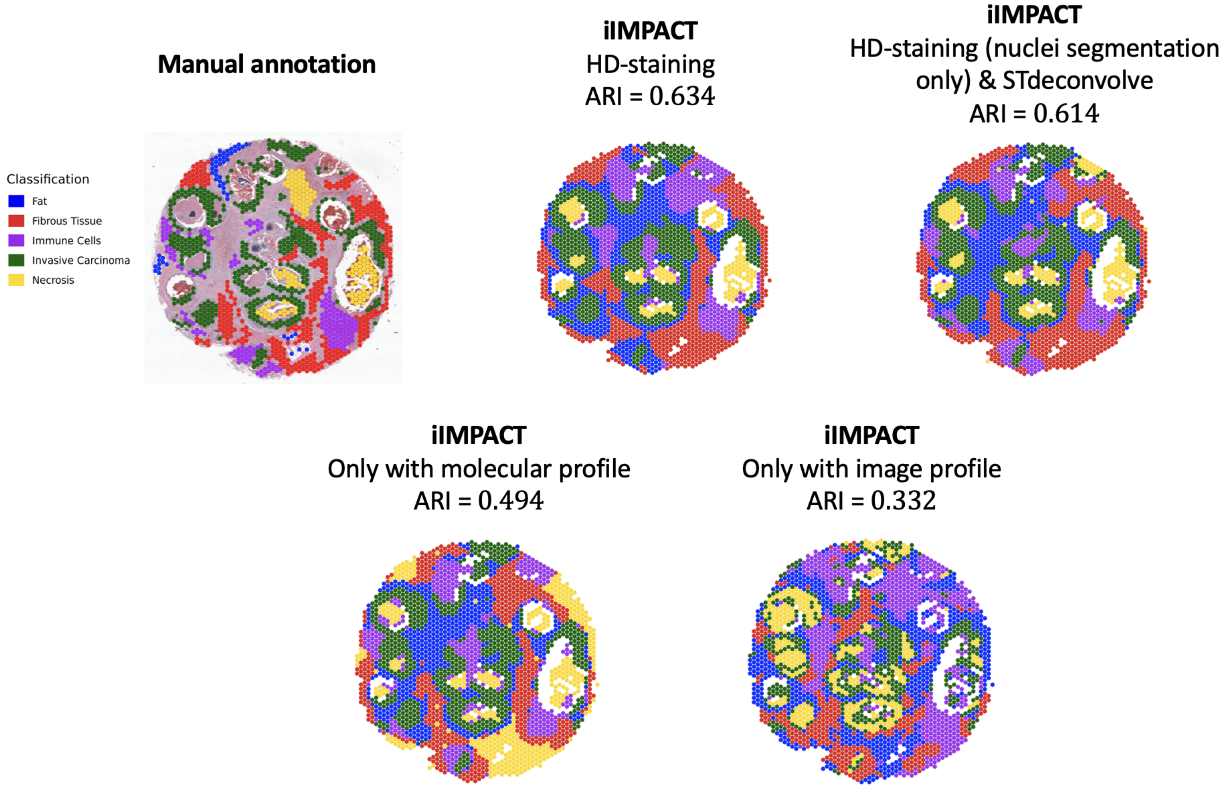

Fig. S15 Human breast cancer data: The matching histology image of the tissue section with manually annotated labels from pathologists, and spatial domains detected by iIMPACT with nuclei identification by HD-staining on the histology image, iIMPACT with nuclei segmentation by HD-staining on the histology image and cell type deconvolution by STdeconvolve on SRT data, iIMPACT assuming the image profile is not available, and iIMPACT assuming the molecular profile is not available, setting the number of spatial domains $K = 5$.

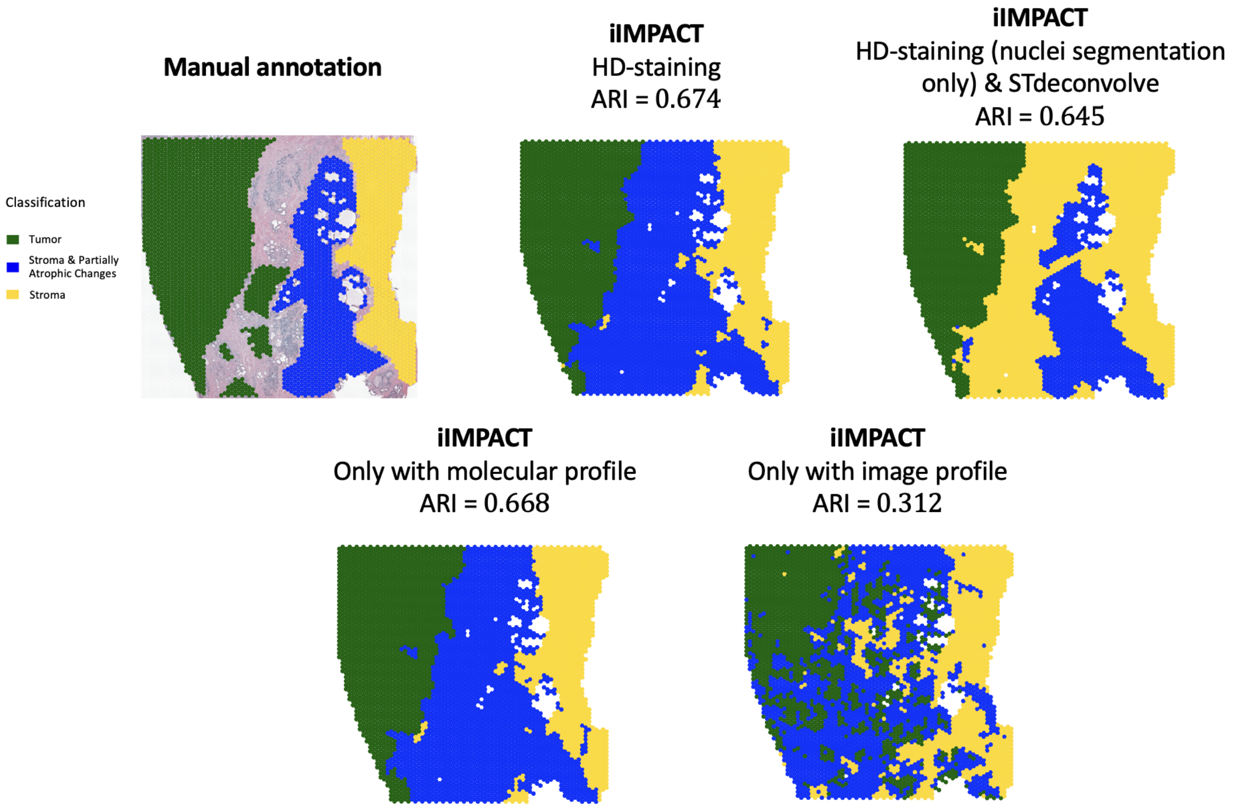

Fig. S16 Human prostate cancer data: The matching histology image of the tissue section with manually annotated labels from pathologists, and spatial domains detected by iIMPACT with nuclei identification by HD-staining on the histology image, iIMPACT with nuclei segmentation by HD-staining on the histology image and cell type deconvolution by STdeconvolve on SRT data, iIMPACT assuming the image profile is not available, and iIMPACT assuming the molecular profile is not available, setting the number of spatial domains $K = 3$.

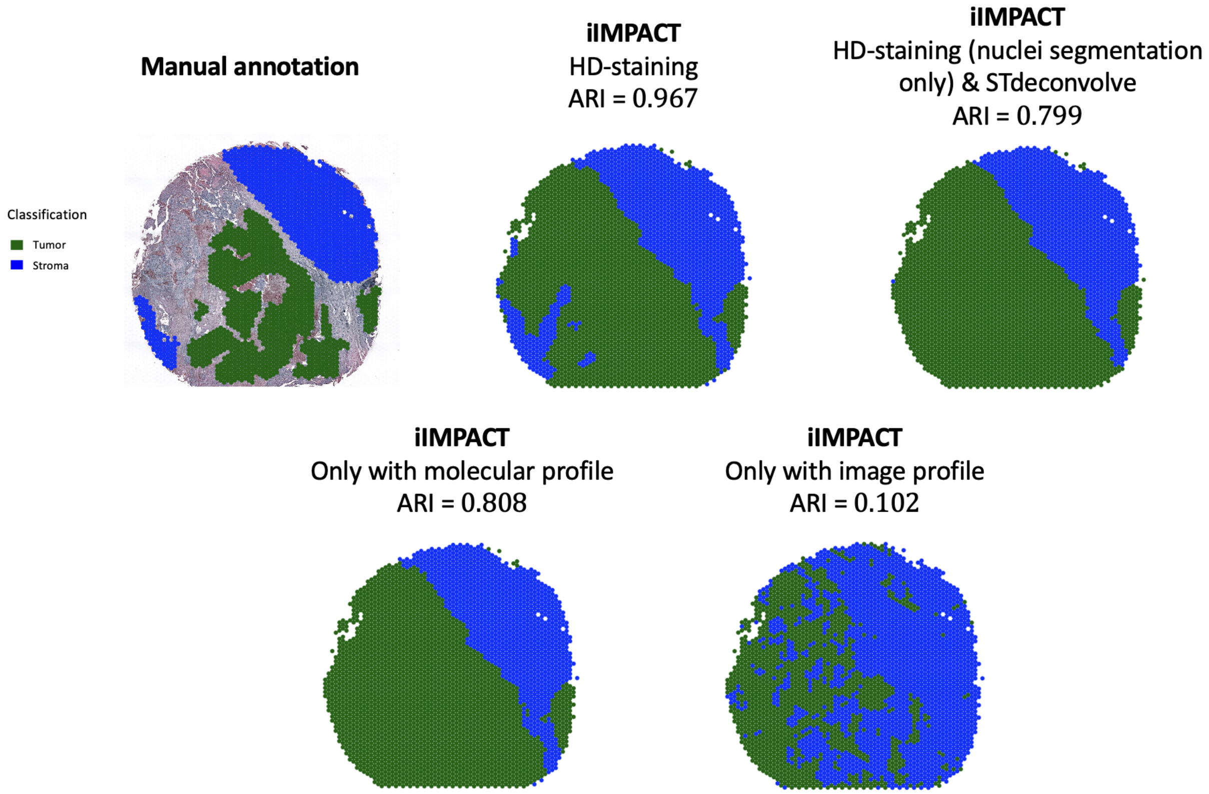

Fig. S17 Human ovarian cancer data: The matching histology image of the tissue section with manually annotated labels from pathologists, and spatial domains detected by iIMPACT with nuclei identification by HD-staining on the histology image, iIMPACT with nuclei segmentation by HD-staining on the histology image and cell type deconvolution by STdeconvolve on SRT data, iIMPACT assuming the image profile is not available, and iIMPACT assuming the molecular profile is not available, setting the number of spatial domains $K = 2$.

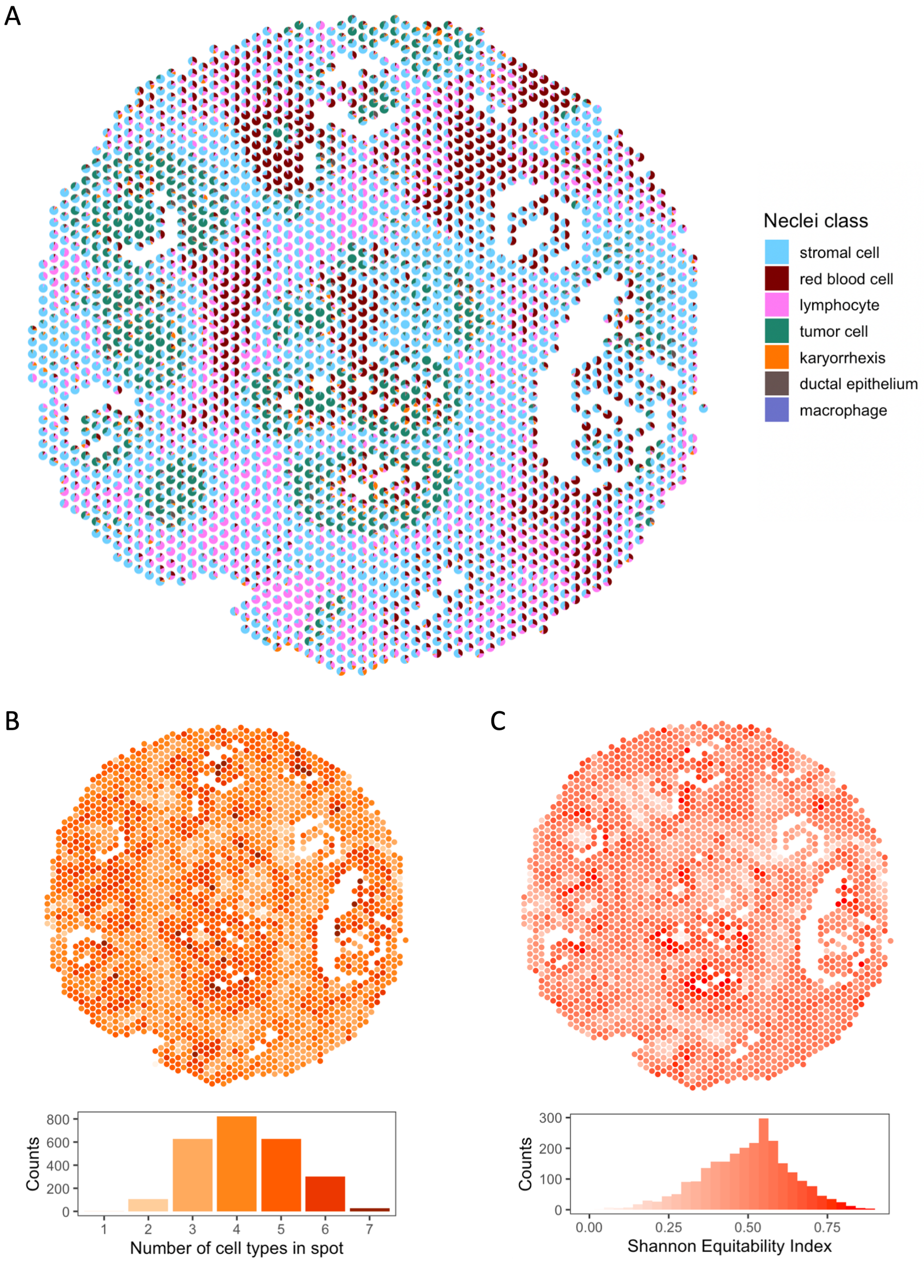

Fig. S18 Human breast cancer data: A. A spatial scatter pie plot displays the cell-type composition on each spot; B. Spatial distribution of number of cell types observed in each spot: Over 70% of spots contain four or more cell types; C. Spatial distribution of Shannon equitability index calculated based on seven cell types for each spot.

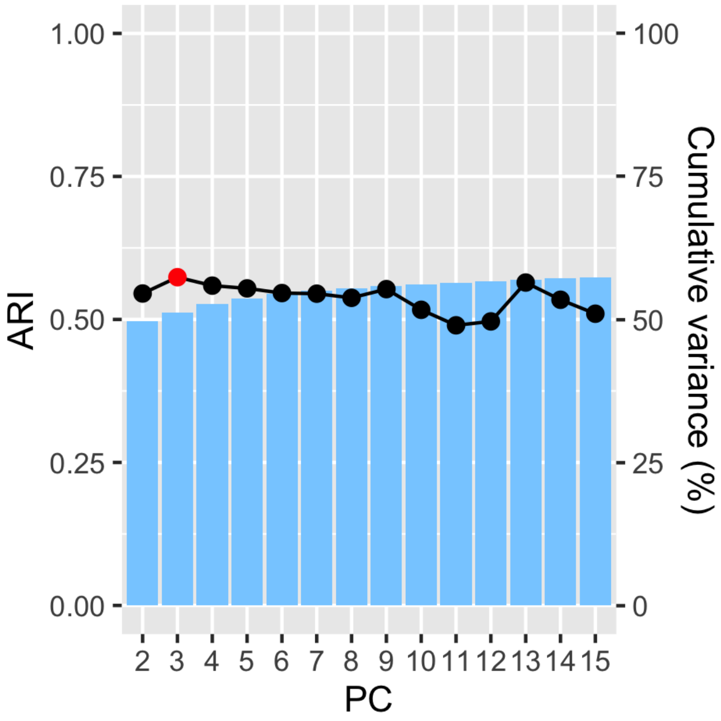

Fig. S19 Sensitivity analysis I: ARIs achieved by iIMPACT when setting the image profile weight w to be zero, and proportion of variance explained under different number of leading principal components in PCA.

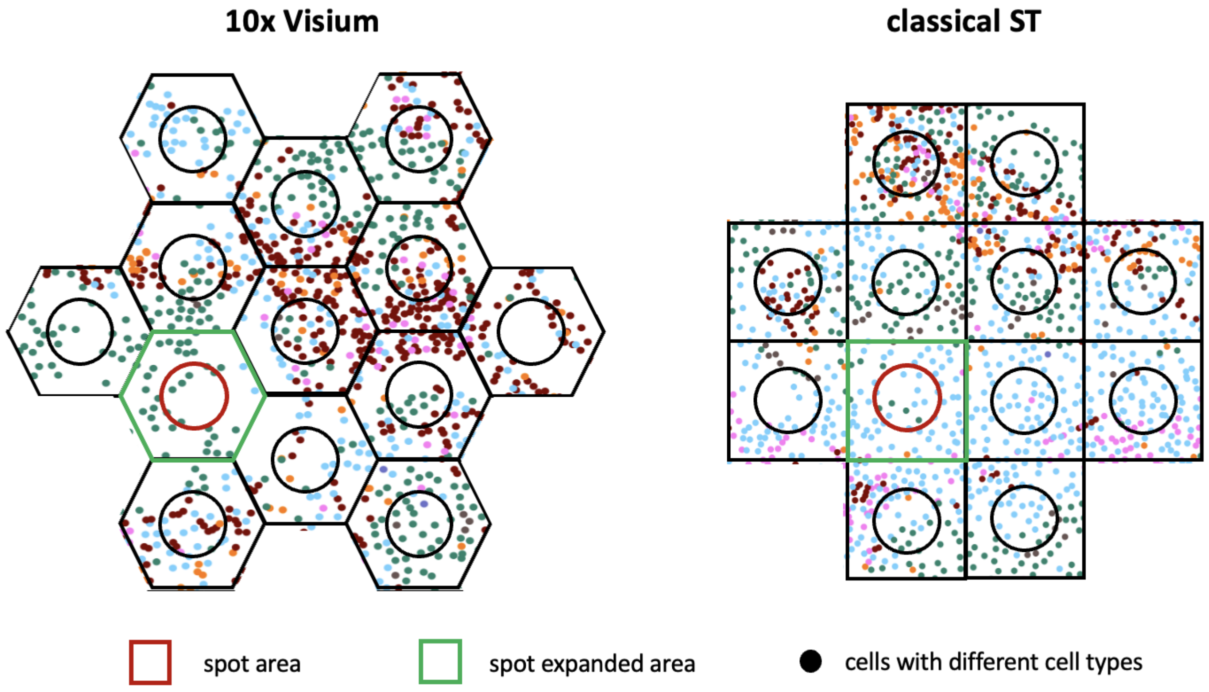

Fig. S20 Demonstration of geometric representations of spatial distribution of spots, and definition of spot expanded area in the 10x Visium and ST technologies.

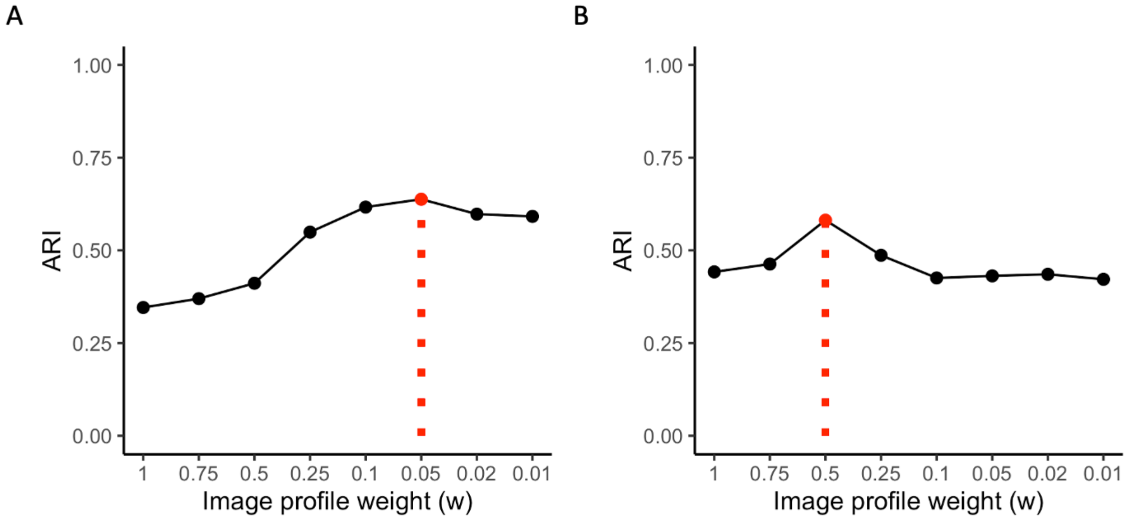

Fig. S21 Sensitivity analysis II: ARIs achieved by iIMPACT clustering under different choices of the image profile weight w on A. human breast cancer data; and B. mouse visual cortex STARmap data.

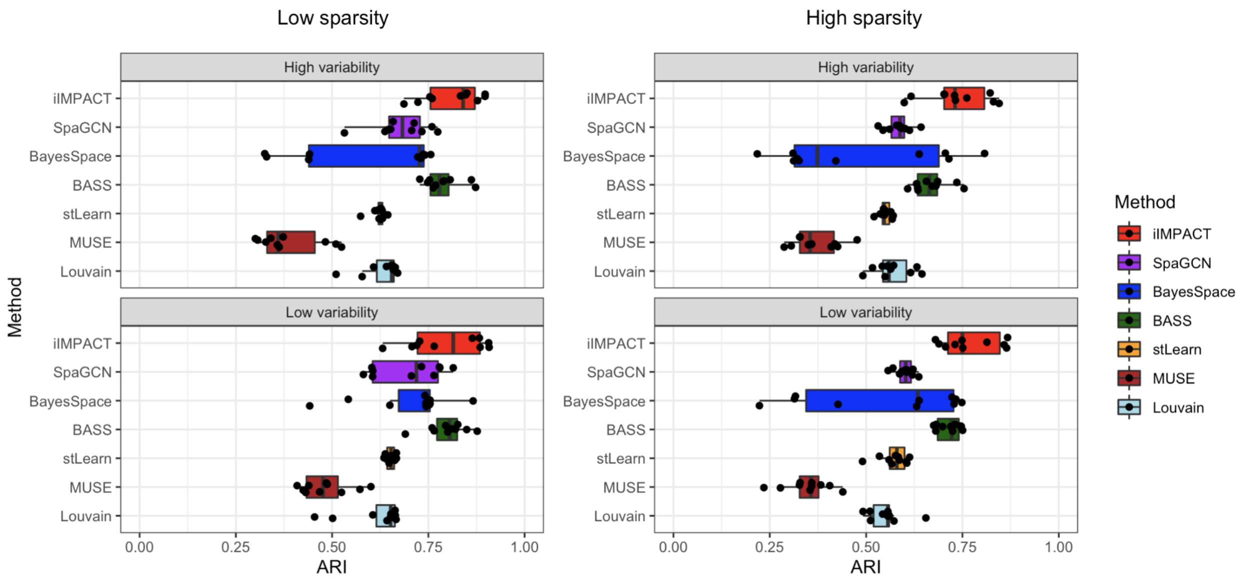

Fig. S22 Simulation study: The boxplots of ARIs achieved by iIMPACT, SpaGCN, BayesSpace, BASS, stLearn, MUSE and Louvain under different scenarios in terms of sparsity and variability settings.

Table S1: Summary of the five real datasets analyzed in the paper.

Dataset name	Technology	Organism	Tissue	Disease	Number of genes	Sample size	Source
Human breast cancer data	ST (10x Visium)	Human	Breast	Ductal carcinoma in situ, invasive carcinoma	17943	2518	10x Genomics
Human prostate cancer data	ST (10x Visium)	Human	Prostate	Adenocarcinoma, invasive carcinoma	17943	4371	10x Genomics
Human ovarian cancer data	ST (10x Visium)	Human	Ovarian	Serous papillary carcinoma	17943	3455	10x Genomics
Mouse visual cortex STARmap data	STARmap	Mouse	Visual cortex	-	1207	1020	[11]
Human DLPFC data	ST (10x Visium)	Human	Dorsolateral prefrontal cortex (DLPFC)	-	33538	4634	[52]

Table S2: Number of genes detected by iIMPACT, SpaGCN, SpatialDE and SPARK, and overlaps with the known gene list for the four real datasets analyzed in the paper.

Dataset	iIMPACT		SpaGCN		SpatialDE		SPARK	
	Number of genes detected	Number of overlaps	Number of genes detected	Number of overlaps	Number of genes detected	Number of overlaps	Number of genes detected	Number of overlaps
Human breast cancer data	1535	112	891	58	1616	91	0	0
Human prostate cancer data	1296	69	1616	72	228	11	0	0
Human ovarian cancer data	1468	98	574	35	2542	163	0	0
Mouse visual cortex STARmap data (Layer: L1)	161	88	127	63	252	123	497	216
Mouse visual cortex STARmap data (Layer: L2/3)	204	59	215	56	252	65	497	132
Mouse visual cortex STARmap data (Layer: L4)	169	73	85	29	252	104	497	165
Mouse visual cortex STARmap data (Layer: L5)	134	47	25	8	252	66	497	107
Mouse visual cortex STARmap data (Layer: L6)	183	80	115	41	252	91	497	152

Table S3: Running time (in minutes) of iIMPACT, SpaGCN, BayesSpace, BASS, stLearn, and MUSE on the five real datasets analyzed in the paper.

Datasets	iIMPACT	SpaGCN	BayesSpace	BASS	stLearn	MUSE
Human breast cancer data	1.78	1.83	18.30	8.53	8.59	28.97
Human prostate cancer data	2.67	2.27	27.99	16.93	32.35	52.35
Human ovarian cancer data	2.24	1.90	23.49	13.22	5.66	41.52
Mouse visual cortex STARmap data	1.09	0.57	13.86	3.60	4.73	-
Human DLPFC data	3.92	2.35	28.92	17.86	6.73	54.49

Table S4: A comparison of spatial domain identification performance among iIMPACT, BayesSpace and BASS where the number of spatial domains K is determined by either experienced pathologists or the integrated completed likelihood (ICL) criterion.

		Number of domains K	Adjusted Rand Index (ARI)		
			iIMPACT	BayesSpace	BASS
Choose K based on pathologists' experience	Human breast cancer data	5	0.634	0.419	0.496
			see the resulting patterns in Fig. 2B		
	Human prostate cancer data	3	0.674	0.522	0.667
			see the resulting patterns in Fig. S8		
	Human ovarian cancer data	2	0.967	0.795	0.794
			see the resulting patterns in Fig. S11		
Choose K based on the ICL	Human breast cancer data	5	0.634	0.419	0.496
	Human prostate cancer data	5	0.626	0.594	0.671
	Human ovarian cancer data	5	0.609	0.590	0.521

Table S5: Spatial domain identification performance (ARIs) of iIMPACT using two data preprocessing strategies, with a comparison with BASS, to three real human cancer data analyzed in the paper.

	Number of domains K	iIMPACT with HD-Staining	iIMPACT with HD-Staining and STdeconvolve	BASS
Human breast cancer data	5	0.634	0.614	0.496
		see the resulting patterns in Fig. S15		Fig. 2
Human prostate cancer data	3	0.674	0.645	0.667
		see the resulting patterns in Fig. S16		Fig. S8
Human ovarian cancer data	2	0.967	0.799	0.794
		see the resulting patterns in Fig. S17		Fig. S11

Table S6: The key notations of the proposed iIMPACT.

	Notation	Support	Definition
Data	N	$N \in \mathbb{N}$	The number of spots
	P	$P \in \mathbb{N}$	The number of genes
	P'	$P' \in \mathbb{N}$, $P' \ll P$	The number of reduced dimensions
	Q	$Q \in \mathbb{N}$	The number of cell types
	K	$K \in \mathbb{N}$	The number of histology-based spatial domains
	$\boldsymbol{C} = [c_{ij}]_{N \times P}$	$c_{ij} \in \mathbb{N}$	The gene expression count table with c_{ij} being the read count for gene j observed at spot i
	$\boldsymbol{Y} = [y_{ij}]_{N \times P'}$	$y_{ij} \in \mathbb{R}$	The low-dimensional gene expression table with y_{ij} being the relative expression on dimension j at spot i
	$\boldsymbol{V} = [v_{iq}]_{N \times Q}$	$v_{iq} \in \mathbb{N}$	The cell type abundance table with v_{iq} being the number of cells with cell type q at spot i and its expanded area
	$\boldsymbol{m} = [m_i]_{N \times 1}$	$m_i \in \mathbb{N}$	The total number of cells at each spot i and its expanded area
	$\boldsymbol{T} = [t_{ir}]_{N \times 2}$	$t_{ir} \in \mathbb{N}$	The x and y coordinates of each spot
	$\boldsymbol{G} = [g_{ii'}]_{N \times N}$	$g_{ii'} \in \{0, 1\}$	The adjacent matrix with $g_{ii'} = 1$ indicating spot i and spot i' are neighbors
	$\boldsymbol{s} = [s_i]_{N \times 1}$	$s_i \in \mathbb{R}^+$	The spot-specific size factors
Model parameters	$\boldsymbol{\Omega} = [\omega_{kq}]_{K \times Q}$	$\omega_{kq} \in [0, 1]$	With ω_{kq} being the relative abundance of cell type q in histology-based spatial domain k
	$\boldsymbol{M} = [\mu_{kj}]_{K \times P'}$	$\mu_{kj} \in \mathbb{R}$	With $\boldsymbol{\mu}_k$ being the mean vector of the normal subcomponent for histology-based spatial domain k
	$\boldsymbol{\Sigma} = [\Sigma_k]_{K \times P' \times P'}$	$\sigma_{kjj'} \in \mathbb{R}^+$	With Σ_k being the covariance matrix of the normal subcomponent for histology-based spatial domain k
	$\boldsymbol{z} = [z_i]_{N \times 1}$	$z_i \in \{1, \ldots, K\}$	The histology-based spatial domain indicators
	$\boldsymbol{\psi} = [\psi_j]_{P \times 1}$	$\psi_j \in \mathbb{R}^+$	The gene-specific dispersion parameters
Tuning parameters	w	$w \in [0, 1]$	Image profile weight

Table S7: Simulation Study: The posterior means of parameters (domain-specific relative abundances of cell types $\hat{\boldsymbol{\Omega}}$ and means of low-dimensional representation of gene expression $\hat{\boldsymbol{M}}$) estimated by iIMPACT on human breast cancer data, which were used in generating the simulated data.

Domain-specific relative abundances of cell types, $\hat{\boldsymbol{\omega}}_1, \hat{\boldsymbol{\omega}}_2, \hat{\boldsymbol{\omega}}_3, \hat{\boldsymbol{\omega}}_4, \hat{\boldsymbol{\omega}}_5$							
	Stromal cell	Karyorrhexis	Lymphocyte	Red blood cell	Tumor cell	Ductal epithelium	Macrophage
Domain 1	0.4209	0.0415	0.0492	0.0905	0.3407	0.0562	0.0009
Domain 2	0.5221	0.0161	0.3346	0.1045	0.0174	0.0045	0.0007
Domain 3	0.2551	0.0141	0.1855	0.5202	0.0166	0.0037	0.0049
Domain 4	0.3875	0.0384	0.0498	0.2802	0.2207	0.0168	0.0066
Domain 5	0.5327	0.0203	0.2502	0.1888	0.0066	0.0011	0.0002

Domain-specific means of low-dimensional representation of gene expression, $\hat{\boldsymbol{\mu}}_1, \hat{\boldsymbol{\mu}}_2, \hat{\boldsymbol{\mu}}_3, \hat{\boldsymbol{\mu}}_4, \hat{\boldsymbol{\mu}}_5$			
	PC 1	PC 2	PC 3
Domain 1	26.2131	2.3886	-0.0389
Domain 2	-2.6583	-5.3326	-1.2944
Domain 3	-6.6993	-2.6915	4.0473
Domain 4	-19.0341	10.3570	1.1856
Domain 5	-18.0996	1.3260	-1.8454
