# Supplementary material for: iIMPACT: integrating image and molecular profiles for spatial transcriptomics analysis
Source: Genome Biol. 2024 Jun 6;25:147. doi: 10.1186/s13059-024-03289-5 (PMC11514947; doi:10.1186/s13059-024-03289-5)
Supplement: Supplementary file 2 — Additional file 2. Review history. [file 13059_2024_3289_MOESM2_ESM.docx]

Review History

**First round of review**

**Reviewer 1**

**Were you able to assess all statistics in the manuscript, including the appropriateness of statistical tests used?**

Yes

**Were you able to directly test the methods?**

Yes

**Comments to author:**

This manuscript describes a new method, iIMPACT, for spatial domain detection and SVG detection in spatial transcriptomics. iIMPACT utilizes a Bayesian normal-multinomial mixture model to integrate the gene expression with cell type information derived from H&E image using HD-staining, and then performs spatial clustering and SVG detection. The paper is clearly written, and I appreciate the large amount of work devoted to this manuscript. However, I am concerned about the novelty of this method and its effectiveness in practice. My main concerns are:

1. One major gap that iIMPACT aims to fill is that many spatial domain detection methods, including Stlearn, BayesSpace, and SpaGCN, cannot fully utilize the morphology information in histology images. The aforementioned methods were published a few years ago, and to my knowledge, many state-of-the-art methods have already addressed this issue. For example, MUSE (https://www.nature.com/articles/s41587-022-01251-z) is capable of integrating spatial gene expression with image features extracted from well-trained Deep NNs, such as ResNet, VIT, and HIPT, to better detect spatial regions. Sigra (https://www.biorxiv.org/content/10.1101/2022.08.18.504464v1) incorporates image feature extraction as one step of the model and simultaneously optimizes it with the spatial domain detection task. The authors should cite and compare their work with these two methods.

2. The utility of iIMPACT appears to be limited due to its heavy reliance on the accurate cell typing obtained through the HD-staining method. HD-staining is a deep-learning model for nuclei segmentation tailored for lung cancer images. Although the authors demonstrate the applicability of this method to other tumor tissue samples, concerns arise regarding its robustness and efficiency. The requirement for precise cell type annotation for each spot severely restricts the practical usability of iIMPACT. In cases where ST data is generated from normal tissue, brain samples, or tumors with overlapping nuclei, obtaining accurate cell type annotations using HD-staining becomes challenging.
Unlike its other methods like BayesSpace and SpaGCN, which can analyze ST data from various tissue types, both with/without H&E images, iIMPACT's usefulness is currently demonstrated only on specific tumor samples. For ST data with low-quality or non-informative H&E images, as well as regions with nuclear overlaps, the segmentation performance and robustness of iIMPACT are questionable. This makes it less practical and more challenging to utilize compared to other available methods.

3. The improvements of iIMPACT over existing method are incremental.
1) Spatial domain separation.
Regarding the breast cancer data (Figure 2B), BayesSpace shows a lower Adjusted Rand Index (ARI) compared to iIMPACT, due to its separation of the tumor into 2 clusters. This observed separation may be a result of tumor heterogeneity and distinct expression patterns within the tumors. It remains uncertain if iIMPACT would also exhibit similar tumor sub-cluster separation when the number of clusters is set to a larger value. Regarding the clustering results for the prostate dataset (Figure 3B) and ovarian dataset (Figure 4B), it is not immediately apparent to me whether iIMPACT outperforms the other two methods. The comparison lacks clear evidence to determine if iIMPACT is superior in these cases. The comparison lacks sufficient evidence to establish iIMPACT's superiority in these cases.
The LIBD dataset (https://www.nature.com/articles/s41593-020-00787-0) is a commonly used benchmark dataset to evaluate spatial domain detection. Since this dataset is generated from human brain with non-informative H&E image, it is difficult to obtain accurate cell type annotation for this dataset. This further reinforces my previous concern on the applicability of iIMPACT in practice.

2) The authors claim that one advantage of iIMPACT is its ability to show the cell type composition of each domain, which other methods cannot do. However, the cell type composition is obtained from another existing method, HD-staining, which I believe is not an original contribution of iIMPACT. Users have the option to employ other spatial domain detection methods and then investigate the cell type composition separately by running HD-staining.

3) The authors claim that one of the contributions of iIMPACT is its ability to identify interactive regions. However, I am skeptical if other methods can achieve the same result. For instance, in Figure 2B, SpaGCN also identified this region, highlighted in red (cluster 5). It raises doubts whether BayesSpace could also identify these regions when the number of clusters is set to be larger.

4. The authors developed a statistically rigorous method for SVG detection in iIMPACT. The authors assert that their method is superior to others by demonstrating its capability to identify more SVGs. However,it is essential to recognize that more identified genes does not necessarily equate to better performance. Not all genes expressed in the COSMIC database should be highly expressed in every tumor ST sample. Additional evidence is required to support the superiority of iIMPACT over other methods. For instance, the genes detected by iIMPACT should exhibit better spatial variability and stronger spatial patterns.

**Reviewer 2**

**Were you able to assess all statistics in the manuscript, including the appropriateness of statistical tests used?**

**Yes:** I find the statistical tests appropriate.

**Were you able to directly test the methods?**

No

**Comments to author:**

This paper describes a statistical framework for identifying spatial domains and the domain-based spatially variable genes for spatial transcriptomics data using three types of inputs: gene expression, spatial proximity, and cell types predicted from histology images, where the usage of the third component is new. An existing method HD-staining is used to predict cell types from histology image. However, there are several points that remain unclear: 1) the general applicability of the method given that the examples currently either rely on a histology-based cell type predictor trained only for cancer cells or known cell type compositions and 2) the reliability of the HD-staining model and its necessarity upon existing cell type deconvolution methods using only gene expression data. Together, this work introduces an interesting and novel way to tackle the challenging task of domain segmentation in ST data and the paper could be much stronger if the above points are addressed. I have several specific comments listed below.

Specific points:
1.The proposed method heavily relies on the high-resolution histology imaging based cell segmentation and cell type prediction by the histology-based digital (HD)-Staining model. The accuracy of HD-Staining model should also be directly validated in the several applications described in the paper.
2.Since the HD-Staining model was trained for cancer cells, it should be clarified what alternatives are to be used for a tissue other than cancer cells without known cell type compositions. If the tool iIMPACT can only handle data related to cancer or data with known cell type compositions, this should be clarified in abstract and/or paper title.
3.As many marker genes are known for various cancer cells, it would improve the utility for users if a quality score of cell type prediction could be provided to the users. For example, the consistency score could be developed to describe the agreement between predicted marker gene expression prediction from histology-based cell type prediction and the gene expression profile from data.
4.The choice of number of domains is briefly mentioned and could be described in more details. Specifically, which of the examples assume a prior knowledge of K? In the examples using a known K, the effectiveness of BIC should be evaluated on its ability to estimate the number of domains.
5.For high-resolution ST data like the starmap example, the data was first coarse-grained and then fed to iIMPACT. Can you directly treat each cell as a spot with a pure cell type composition and apply iIMPACT without lowering the spatial resolution of the data used?
6.As stLearn is a highly related method which also uses histology images though in a different way, a comparison to it should be added, for example, clustering result using both gene expression and the morphological features obtained using stlearn.pp.extract_feature.
7.While using histology-based cell type prediction in domain segmentation for ST data is a novel approach, benifit of this approach need to be demonstrated. Does HD-Staining improve upon using existing cell type deconvolution approaches such as "Miller, Brendan F., et al. "Reference-free cell type deconvolution of multi-cellular pixel-resolution spatially resolved transcriptomics data." Nature communications 13.1 (2022): 2339."?

**Reviewer 3**

**Were you able to assess all statistics in the manuscript, including the appropriateness of statistical tests used?**

**Yes:** See comments

**Were you able to directly test the methods?**

No

**Comments to author:**

While spatial transcriptomics has enabled us to characterize gene expression while preserving spatial organizing within tissues, current clustering analysis of such gene expression profiles do not utilize histology images to incorporate morphological features alongside molecular information even though this could improve accuracy of cluster partitioning. In this manuscript, the authors developed a method, iIMPACT, that uses AI-reconstructed histology images to define histology-based spatial domains (neighboring cells with similar morphological and molecular profiles) through a Bayesian finite mixture model and to identify domain-specific spatially variable genes through a negative binomial regression model. The authors claim that iIMPACT achieved higher accuracy at identifying spatial domains when compared to manually annotated domains by pathologists than existing methods such as BayesSpace and SpaGCN that integrate image features like RGB color values. Among its unique capabilities, the authors claim iIMPACT identifies interaction zones with unique transcriptional profiles. While we found the interaction-zone analyses particularly interesting, we have the following major and minor comments:

Major comments:

- To evaluate performance, the authors compare spatial domains derived from evaluated methods with those derived manually from pathologist. However, pathology annotations are based on only histological information and not molecular information. Any method that integrates both histological and molecular information should presumably identify features that are not perfectly consistent with features derived from just histology. If the authors disregard all molecular information and just use histological information, do they achieve a higher correspondence with pathology annotations? Such a negative control should be included.

- Given that many of these methods are sensitive to parameters, what if the number of domains was decreased? Could the accuracy increase? In general, sensitivity to parameters must be evaluated.

- More conceptually, what if the tumor region does have two sub populations that are molecular distinct or even morphologically but the pathologists were not asked to differentiate this when making their manual annotations? More broadly, should pathologist annotations of histology images be used as ground truth for methods that incorporate molecular information to do classification? A discussion of this is warranted.

- Likewise, the authors quantified performance based on adjusted rand index for evaluating consistency between spatial domains and manual annotations. While iIMPACT appears to have done better (ARI = 0.634) compared to other methods (ARI=0.520 for SpaGCN and ARI = 0.419 for BayeSpace), these seem all to be quite poor performance. What is a baseline level of performance and does a ~0.1 ARI indicate a substantial and significant improvement?

- In Fig 2,3,4F, SPARK has 0 overlap. Is this because SPARK failed to run? Or because it really gave results that had no overlap?

- In the breast cancer data evaluated, fat tissue doesn't seem to be represented in any of the domains identified by iIMPACT or other methods (pg 8 line 27). Does that mean you would not expect to recapitulate a fat spatial domain? This limitation should be discussed along with any other cell-type or domain-specific limitations.

- We were unclear as to what is the advantage of using cell types from HD-Staining to interpret domains instead of using molecular information?

- The authors suggest that "identified zones [have] a high level of heterogeneity in gene expression and complex cellular interactions" seems like an excessive claim (p9 line 42). We were unsure whether the level of heterogeneity that exists in these interaction zones were any more significant than the result of profiling spots that have a mix of two cell types. The authors need to be able to differentiate if this "gene expression richness" is not just the result of mixing two cell types and instead the result of complex cellular interactions to support their claim.

- The analysis of domain-specific SVGs here seems to confound ideas of SVGs (genes with spatial autocorrelation) with differentially expressed genes (DEGs) between domain. In the methods, the authors define SVGs as "differentially expressed in a given histology-based spatial domain 𝑘 compared with all other domains." (p 27 line 26) The authors do not describe how they used SpaGCN, SpatialDE, and SPARK to get SVGs so it is not clear if these are actually SVGs or also DEGs. We suggest the authors replace uses of SVG with DEG between domains where appropriate.

- Similar to ARI, we had a difficult time interpreting the fold enrichment (odds ratio) graphs and how it showed that "compared with other SVG detection methods, iIMPACT-defined SVGs are more enriched of known functional genes" and support the claim that "iIMPACT could provide a better understanding of both cellular spatial organization and functional gene landscape of developmental and diseased tissues." (p 15 line 39). It is not clear to us what a baseline level of performance would be and whether iIMPACT's 1.7x10^-14 represents a substantial and significant improvement over SpaGCN at 8.0 x 10^-10 for example.

- Overall, it is not clear the sensitivity of iIMPACT as an analysis method. The authors claim that iIMPACT identifies genes that are interpretable. But how many of these are false positives? For example, the authors analyzed 1020 genes in STARmap (p 13) and describe two iIMPACT-defined SVGs as being functionally relevant. How many genes did iIMPACT identify? Just these two?

Minor comments:

- The authors describe spatially resolved transcriptomics as a "new generation of RNA sequencing analysis techniques". We find this description to mischaracterize imaging-based spatial transcriptomics technologies which are a new generation of smFISH not sequencing. We believe "new generation of RNA profiling techniques" would be more appropriate.

- The authors claim that image features from as stLearn, hidden-Markov random field, BayesSpace, and SpaGCN "fail to provide biologically relevant insight" (p4 line 12). We believe this is an overstatement and very dismissive of prior work that have already been used to by others to derive biological insights.

- The authors claim that their tool is uniquely able to "interpret the identified domains in a biological meaningful way" (p 9 l4) but this is something that people usually do downstream of clustering into domains or cell type by using tools for differential gene expression analysis.

- We did not find the genes visualized in Fig 5D to be compelling as SVGs. Perhaps an alternative visualization would be better.

- The paper would benefit from explicitly defining AI-reconstructed (p4 line 41).

**Authors Response**

**Point-by-point responses to the reviewers’ comments:**

We appreciate the editor and the three referees for allowing us to refine our manuscript. Based
on their insightful feedback, we have conducted a substantial revision of our manuscript. The guidance provided by the referees was instrumental in enhancing the quality of our work. We hope the revised manuscript is now acceptable for publication in *Genome Biology*. Below, we respond to the editor and each referee's comments, making references to specific sections, figures, and tables in both the revised main text and supplementary materials. For ease of review, we have highlighted all changes to the main text and supplementary materials since our initial submission in blue.

**Editor: Comments and Responses**

**Comment 1**: *Referees 1 and 3 have raised concerns about the absence of comprehensive citations and rigorous comparisons and evaluations with existing methodologies. It is unclear at the moment if the proposed method provides substantial improvement upon existing methods, both in cancer tissues and beyond cancer tissue types.*

**Response:** We agree with the editor that an expanded comparative analysis is needed. To this end, we have incorporated evaluations of three additional, recently developed spatial domain identification methods: BASS [1], MUSE [2], and stLearn [3]. For iIMPACT and each comparative method, we have assessed their clustering efficacy across various numbers of spatial domains $K$, as shown in Figure S4 in the supplementary materials (attached below), and have visualized their corresponding spatial domain patterns in Figures S3, S9, and S12 in the supplementary materials. Furthermore, to demonstrate iIMPACT's applicability in spatial domain identification beyond cancer tissues, we have analyzed the LIBD human dorsolateral prefrontal cortex (DLPFC) data [4], which was obtained using the 10x Visium platform from normal brain tissues. The findings from this additional data analysis are detailed in Section S3, with a graphical summary provided in Figure S14 (attached below), both of which are included in the supplementary materials.

*Figure S4: ARIs achieved by six spatial domain identification methods, iIMPACT, SpaGCN, BayesSpace, BASS, stLearn, and MUSE, under different choices of the number of spatial domains*$K$ *on the A. human breast cancer data; B. human prostate cancer data; and C. human ovarian cancer data.*

*Figure S14: LIBD human dorsolateral prefrontal cortex (DLPFC) data: The matching histology image of the tissue section with manually annotated labels from the original study, and histology-based spatial domains detected by iIMPACT, SpaGCN, BayesSpace, BASS, stLearn, and MUSE, setting the number of spatial domains* $K=7$*.*

**Comment 2**: *Both Referees 1 and 2 have also raised concerns about the method’s heavy reliance on precise cell typing, which may or may not be obtainable in practice.*

**Response**: We acknowledge the referees' point on the limitations associated with the cell typing method employed. Nuclei segmentation and classification are essential processes in histology image analysis. In response to the referees’ concerns, we have incorporated an in-depth discussion (Line 1 on Page 17, attached below) in the Discussion section. Furthermore, we have outlined an alternative data preparation pipeline in Figure S13 (attached below), which relies on nuclei segmentation (e.g., Hover-Net [5]) and utilizes reference-free cell-type deconvolution methods (e.g., STdeconvolve [9]). This pipeline is particularly advantageous in cases where precise cell typing is impractical. To validate the utility of this alternative data preprocessing of iIMPACT, we have conducted a new analysis on the LIBD human DPLFC data. The details of this case study are reported in Section S3 of the supplementary materials, with a graphical summary provided in Figure S14.

Page 17, Line 1:

*Nuclei identification methods for histology image analysis exhibit several limitations that hinder their widespread applicability and accuracy. One primary challenge is their generalizability. Most deep-learning-based algorithms require model training on high-quality labeled data, making them less adaptable to varied datasets and potentially limiting their generalizability across different tissue types and staining techniques. Besides, the performance of these nuclei identification methods may decrease when handling overlapping nuclei, where segmentation becomes intricate due to the lack of clear boundaries. To address the limitations of existing nuclei identification methods and enhance the versatility of iIMPACT, we proposed an alternative approach for the data preparation outlined in Figure S13 of the supplementary materials. When implementing iIMPACT on tissue sections where precise nuclei classification proves challenging using nuclei identification methods, we leveraged the outputs from deep-learning-based or statistical nuclei segmentation methods to derive the nuclei localization, enabling us to determine the number of nuclei in each spot. Many methods [6-8] exist for isolating cell nuclei across various tissue types without relying on manual labeling data for training. Subsequently, we recommend using reference-free cell-type deconvolution methods [9] to generate the cell type abundance table. This data preparation pipeline was applied to additional SRT data, the LIBD human dorsolateral prefrontal cortex (DLPFC) data generated via 10x Visium [4]. Notably, iIMPACT demonstrated superior performance in spatial domain identification under this alternative approach to generate the image profile (Figure S14 in the supplementary materials). Details are introduced in Section S3 of the supplementary materials. We also validated this data preparation pipeline on the human breast cancer data, and iIMPACT achieved better performance than only utilizing the molecular profile, as shown in Figure S15 in the supplementary materials.*

*Figure S13: An alternative pipeline for data preparation: Reference-free cell type deconvolution algorithms are applied on the molecular profile to generate the cell type abundance table, together with the nuclei localization via nuclei identification methods.*

**Comment 3**: *Furthermore, upon closer examination, we have identified methodological similarities between the proposed method and a previously published work, BASS, within the same journal. Therefore, it is important that the revised manuscript includes a detailed discussion on how the proposed method distinguishes itself from BASS and a thorough comparison thereof in all datasets.*

**Response**: We apologize for the oversight in not referencing BASS, a pioneering work in multi-sample SRT data analysis. In response to the editor's comment, we have now elaborated on the distinct aspects of iIMPACT in contrast to BASS within the Discussion section (Line 10 on Page 16, attached below). Additionally, we have included BASS in our simulation study and across all real data analyses (see the graphical summary in Figures 2, 3, 4, and 5 of the main text and in Figures S3, S4, S8, S9, S11, S12, S14, and S19 of the supplementary materials). For the editor's convenience, we outline the five key distinctions between iIMPACT and BASS below:

1. BASS is tailored for multi-sample analysis, in contrast, the current version of iIMPACT is optimized for spatial domain identification within single-sample SRT data.
2. BASS is primarily designed for single-cell resolution SRT data analysis derived from imaging-based SRT techniques, while iIMPACT is developed for NGS-based SRT data analysis at the spot resolution. It is noteworthy that both methods are flexible enough to handle the converse data type using the concepts of "pseudo" cells or "pseudo" spots.
3. iIMPACT integrates both image and molecular profiles for spatial domain identification. However, BASS does not utilize information from the paired histology image.
4. Regarding model design, iIMPACT assumes independence between cell type abundance and gene expression profiles within a given spatial domain. In contrast, BASS postulates that gene expression profiles are conditional on the cell type composition, which in turn depends on the spatial domain.
5. In the analysis of NGS-based SRT data at spot resolution, BASS estimates the cell type composition for each individual spot, whereas iIMPACT dedicates its analysis to deciphering cell type composition for each spatial domain.

Page 16, Line 10:

*iIMPACT, BASS, and BayesSpace utilize a Bayesian mixture model with a Markov random field model for the identification of smooth spatial domains based on the SRT molecular profile. Unlike BASS and BayesSpace omit complementary information from the paired histology image, iIMPACT integrates cell type abundance derived from the image as an additional component. This integration significantly improves the accuracy of spatial domain identification and enables the biological interpretability of these domains. Moreover, iIMPACT assumes the image and molecular profiles—specifically, cell type abundance and gene expression levels—to contribute to spatial clustering, with adjustable weighting to optimize results. In contrast, BASS models cell type composition as a hidden layer within its Bayesian hierarchical model, asserting a direct probabilistic link between gene expression features and latent cell types. Notably, BASS specializes in analyzing imaging-based SRT data, which typically achieves the single-cell resolution and supports multi-sample clustering, whereas iIMPACT conducts spatial domain identification at the spot resolution, rendering it more suitable for analyzing NGS-based SRT data.*

**Referee 1: Comments and Responses**

**Comment 1**: *This manuscript describes a new method, iIMPACT, for spatial domain detection and SVG detection in spatial transcriptomics. iIMPACT utilizes a Bayesian normal-multinomial mixture model to integrate the gene expression with cell type information derived from H&E image using HD-staining, and then performs spatial clustering and SVG detection. The paper is clearly written, and I appreciate the large amount of work devoted to this manuscript.*

**Response**: We appreciate the referee's positive feedback and insightful comments. These have greatly helped us to improve the quality of our manuscript.

**Comment 2**: *One major gap that iIMPACT aims to fill is that many spatial domain detection methods, including stLearn, BayesSpace, and SpaGCN, cannot fully utilize the morphology information in histology images. The aforementioned methods were published a few years ago, and to my knowledge, many state-of-the-art methods have already addressed this issue. For example, MUSE is capable of integrating spatial gene expression with image features extracted from well-trained Deep NNs, such as ResNet, VIT, and HIPT, to better detect spatial regions. Sigra incorporates image feature extraction as one step of the model and simultaneously optimizes it with the spatial domain detection task. The authors should cite and compare their work with these two methods.*

**Response**: We agree with the referee's recommendation to incorporate comparisons with recent methodologies. Accordingly, we have expanded our analysis to include MUSE [2], BASS [1], and stLearn [3] for all analyzed datasets, including the newly included LIBD human DLPFC data. These additional comparisons have substantiated the robustness of our iIMPACT (see Figures 2, 3, 4, and 5 of the main text and Figures S3, S4, S8, S9, S11, S12, S14, and S19 of the supplementary materials). SiGra was not included in our comparative analysis due to technical challenges encountered during code implementation.

**Comment 3**: *The utility of iIMPACT appears to be limited due to its heavy reliance on the accurate cell typing obtained through the HD-staining method. HD-staining is a deep-learning model for nuclei segmentation tailored for lung cancer images. Although the authors demonstrate the applicability of this method to other tumor tissue samples, concerns arise regarding its robustness and efficiency. The requirement for precise cell type annotation for each spot severely restricts the practical usability of iIMPACT. In cases where ST data is generated from normal tissue, brain samples, or tumors with overlapping nuclei, obtaining accurate cell type annotations using HD-staining becomes challenging. Unlike its other methods like BayesSpace and SpaGCN, which can analyze ST data from various tissue types, both with/without H&E images, iIMPACT's usefulness is currently demonstrated only on specific tumor samples. For ST data with low-quality or non-informative H&E images, as well as regions with nuclear overlaps, the segmentation performance and robustness of iIMPACT are questionable. This makes it less practical and more challenging to utilize compared to other available methods.*

**Response**: We acknowledge the referee's point on the limitations associated with the cell typing method employed. Nuclei segmentation and classification are essential processes in histology image analysis. In response to the referee's concern, we have incorporated an in-depth discussion (Line 1 on Page 17, attached below) in the Discussion section. Furthermore, we have outlined an alternative data preparation pipeline in Figure S13 (attached below), which relies on nuclei segmentation (e.g., Hover-Net [5]) and utilizes reference-free cell-type deconvolution methods (e.g., STdeconvolve [9]). This pipeline is particularly advantageous in cases where precise cell typing is impractical. To validate the effectiveness of this alternative data processing strategy, we have conducted additional analyses on the human breast cancer data and the newly included LIBD human DPLFC data. The details of the human DPLFC case study are described in Section S3 of the supplementary materials. The graphical summaries of these two case studies are presented in Figure S15 (attached below) and Figure S14, respectively. The resulting ARIs were consistently higher than those of the competing methods. Addressing the referee’s concern regarding iIMPACT’s potential underperformance when analyzing SRT data accompanied by low-quality or non-informative H&E images, we assessed iIMPACT without using the image profile on the human breast cancer data. The findings, presented in Figure S15 (attached below) of the supplementary materials, demonstrate that iIMPACT still maintained a competitive spatial domain identification performance (ARI$=0.494$) relative to other methods.

Page 17, Line 1:

*Nuclei identification methods for histology image analysis exhibit several limitations that hinder their widespread applicability and accuracy. One primary challenge is their generalizability. Most deep-learning-based algorithms require model training on high-quality labeled data, making them less adaptable to varied datasets and potentially limiting their generalizability across different tissue types and staining techniques. Besides, the performance of these nuclei identification methods may decrease when handling overlapping nuclei, where segmentation becomes intricate due to the lack of clear boundaries. To address the limitations of existing nuclei identification methods and enhance the versatility of iIMPACT, we proposed an alternative approach for the data preparation outlined in Figure S13 of the supplementary materials. When implementing iIMPACT on tissue sections where precise nuclei classification proves challenging using nuclei identification methods, we leveraged the outputs from deep-learning-based or statistical nuclei segmentation methods to derive the nuclei localization, enabling us to determine the number of nuclei in each spot. Many methods [6-8] exist for isolating cell nuclei across various tissue types without relying on manual labeling data for training. Subsequently, we recommend using reference-free cell-type deconvolution methods [9] to generate the cell type abundance table. This data preparation pipeline was applied to additional SRT data, the LIBD human dorsolateral prefrontal cortex (DLPFC) data generated via 10x Visium [4]. Notably, iIMPACT demonstrated superior performance in spatial domain identification under this alternative approach to generate the image profile (Figure S14 in the supplementary materials). Details are introduced in Section S3 of the supplementary materials. We also validated this data preparation pipeline on the human breast cancer data, and iIMPACT achieved better performance than only utilizing the molecular profile, as shown in Figure S15 in the supplementary materials.*

*Figure S13: An alternative pipeline for data preparation: Reference-free cell type deconvolution algorithms are applied on the molecular profile to generate the cell type abundance table, together with the nuclei localization via nuclei identification methods.*

*Figure S15: Human breast cancer data: The matching histology image of the tissue section with manually annotated labels from pathologists, and spatial domains detected by iIMPACT with nuclei identification by HD-staining on the histology image, iIMPACT with nuclei segmentation by HD-staining on the histology image and cell type deconvolution by STdeconvolve on SRT data, and iIMPACT assuming the image profile is not available, setting the number of spatial domains to be* $K=5$*.*

**Comment 4**: *The improvements of iIMPACT over existing method are incremental.*

*1) Spatial domain separation: Regarding the breast cancer data (Figure 2B), BayesSpace shows a lower Adjusted Rand Index (ARI) compared to iIMPACT, due to its separation of the tumor into 2 clusters. This observed separation may be a result of tumor heterogeneity and distinct expression patterns within the tumors. It remains uncertain if iIMPACT would also exhibit similar tumor sub-cluster separation when the number of clusters is set to a larger value. Regarding the clustering results for the prostate dataset (Figure 3B) and ovarian dataset (Figure 4B), it is not immediately apparent to me whether iIMPACT outperforms the other two methods. The comparison lacks clear evidence to determine if iIMPACT is superior in these cases. The comparison lacks sufficient evidence to establish iIMPACT's superiority in these cases. The LIBD dataset is a commonly used benchmark dataset to evaluate spatial domain detection. Since this dataset is generated from human brain with non-informative H&E image, it is difficult to obtain accurate cell type annotation for this dataset. This further reinforces my previous concern on the applicability of iIMPACT in practice.*

*2) The authors claim that one advantage of iIMPACT is its ability to show the cell type composition of each domain, which other methods cannot do. However, the cell type composition is obtained from another existing method, HD-staining, which I believe is not an original contribution of iIMPACT. Users have the option to employ other spatial domain detection methods and then investigate the cell type composition separately by running HD-staining.*

*3) The authors claim that one of the contributions of iIMPACT is its ability to identify interactive regions. However, I am skeptical if other methods can achieve the same result. For instance, in Figure 2B, SpaGCN also identified this region, highlighted in red (cluster 5). It raises doubts whether BayesSpace could also identify these regions when the number of clusters is set to be larger.*

**Response**:

1) We recognize the referee's concerns regarding the evaluation of iIMPACT's performance on the human breast cancer data. In response, we have applied iIMPACT and the other methods to all analyzed cancer data under different numbers of spatial domains $K=2,\cdots,8$, and presented the resulting spatial domain patterns in Figures S3, S9, and S12 of the supplementary materials (attached below). For the human breast cancer data, BayesSpace delineated the tumor regions into two distinct clusters, indicated in red and green. Notably, BayesSpace identified two separate components, one centrally located at the lower part and another to the right. Even as the number of clusters $K$ increased, iIMAPCT, SpaGCN, and BASS consistently grouped the central lower region with the other tumor regions, highlighting a divergence from BayesSpace’s findings. Concerning the prostate and ovarian cancer data, we assessed the clustering outcomes against manual annotations by Dr. Liwei Jia, an experienced pathologist and co-author of this manuscript. These comparisons, showcased in Figures S8 and S11 of the supplementary materials (attached below), underscore the satisfactory performance of iIMPACT. Moreover, for a quantitative assessment, we calculated the ARIs between the clustering results and the manual annotations for varying $K$, shown in Figure S4 in the supplementary materials (attached below).

*Figure S3: Human breast cancer data: The histology-based spatial domains detected by iIMPACT, SpaGCN, BayesSpace, BASS, stLearn, and MUSE, setting the number of spatial domains* $K$ *from 2 to 8.*

*Figure S9: Human prostate cancer data: The histology-based spatial domains detected by iIMPACT, SpaGCN, BayesSpace, BASS, stLearn, and MUSE, setting the number of spatial domains* $K$ *from 2 to 8.*

*Figure S12: Human ovarian cancer data: The histology-based spatial domains detected by iIMPACT, SpaGCN, BayesSpace, BASS, stLearn, and MUSE, setting the number of spatial domains* $K$ *from 2 to 8.*

*Figure S8: Human prostate cancer data: The matching histology image of the tissue section with manually annotated labels from pathologists, and spatial domains detected by iIMPACT, SpaGCN, BayesSpace, BASS, stLearn, and MUSE, setting the number of spatial domains to be* $K=3$*.*

*Figure S11: Human ovarian cancer data: The matching histology image of the tissue section with manually annotated labels from pathologists, and histology-based spatial domains detected by iIMPACT, SpaGCN, BayesSpace, BASS, stLearn, and MUSE, setting the number of spatial domains to be* $K = 2$*.*

*Figure S4: ARIs achieved by six spatial domain identification methods, iIMPACT, SpaGCN, BayesSpace, BASS, stLearn, and MUSE, under different choices of the number of spatial domains*$K$ *on the A. human breast cancer data; B. human prostate cancer data; and C. human ovarian cancer data.*

2) We acknowledge the possibility of using alternative spatial domain detection methods followed by separate cell-type composition analysis through HD-staining. However, the novelty of iIMPACT is its integrated approach, which enables the identification of spatial domains, while estimating their respective cell type relative abundances simultaneously. This joint modeling approach typically outperforms multi-stage methods, which can yield sub-optimal results. We have made this advantage more explicit in the revised main text, specifically on Page 9, Line 3.

Page 9, Line 3:

*Secondly, iIMPACT is able to define each individual histology-based spatial domain by inferring the latent spatial domain-specific relative abundance of cell types parametrized by the Bayesian multinomial-normal mixture model (Figure 2C).*

3) We acknowledge that while alternative methods may identify interactive regions, iIMPACT's novel contribution lies in its ability to rigorously define such regions only by quantifying the uncertainty of assigning spots into different spatial domains. In contrast, other methods need additional analysis to achieve the same results. To improve clarity, we have revised the relevant parts of the main text.

Page 9, Line 16:

*Interactive zones are distinguished from the identified spatial domain. It is defined as spots with higher uncertainty on domain allocation, which potentially have higher diversity in cell type abundance and heterogeneity in gene expression compared with neighboring spots with unambiguous domain definition.*

**Comment 4**: *The authors developed a statistically rigorous method for SVG detection in iIMPACT. The authors assert that their method is superior to others by demonstrating its capability to identify more SVGs. However, it is essential to recognize that more identified genes do not necessarily equate to better performance. Not all genes expressed in the COSMIC database should be highly expressed in every tumor ST sample. Additional evidence is required to support the superiority of iIMPACT over other methods. For instance, the genes detected by iIMPACT should exhibit better spatial variability and stronger spatial patterns.*

**Response**: We agree with the referee that more evaluations for the spatial domain-specific differentially expressed genes (spaDEGs) identified by iIMPACT should be included. Beyond the initial analysis, we have computed the Moran's I [10], a commonly used metric for measuring spatial autocorrelation, for all spaDEGs identified by iIMPACT and SVGs detected by SpaGCN, SPARK, and SpatialDE. Results are presented in Figure S5 in the supplementary materials (attached below). The violin plot comparison demonstrates that spaDEGs identified by iIMPACT exhibit a higher average Moran's I value, signifying more pronounced spatial autocorrelation. We have added the related discussion in the main text (Page 10, Line 16) and Section S1 of the supplementary materials.

Page 10, Line 16:

*To have an additional diagnosis of the spatial signals of those detected genes, we employed the Moran’s I [10] statistic to quantify the degree of spatial autocorrelation of gene expression (Details in Section S1 of the supplementary materials). Results are shown in Figure S5 in the supplementary materials. Genes detected by iIMPACT exhibit a notably higher average Moran’s I than those detected by SpaGCN and SPARK across various selection thresholds. Additionally, these genes demonstrate a higher average Moran’s I than the top* $1,000$ *SpatialDE-identified SVGs.*

*Figure S5: Evaluation of spaDEGs detected by iIMPACT: The average Moran’s I’s for the spaDEGs/SVGs detected by four methods (i.e., iIMPACT, SpaGCN, SpatialDE, and SPARK) under the setting of six selection thresholds of top genes, and the violin plots of Moran’s I’s of top 1000 spaDEGs/SVGs detected by four methods, for the A. human breast cancer data; B. human prostate cancer data; and C. human ovarian cancer data.*

**Referee 2: Comments and Responses**

**Comment 1**: *This paper describes a statistical framework for identifying spatial domains and the domain-based spatially variable genes for spatial transcriptomics data using three types of inputs: gene expression, spatial proximity, and cell types predicted from histology images, where the usage of the third component is new. An existing method, HD-staining, is used to predict cell types from histology images. However, there are several points that remain unclear: 1) the general applicability of the method given that the examples currently either rely on a histology-based cell type predictor trained only for cancer cells or known cell type compositions. And 2) the reliability of the HD-staining model and its necessity upon existing cell type deconvolution methods using only gene expression data. Together, this work introduces an interesting and novel way to tackle the challenging task of domain segmentation in ST data, and the paper could be much stronger if the above points are addressed.*

**Response**: We appreciate the referee's positive feedback and have addressed each of his/her comments as detailed below.

**Comment 2**: *The proposed method heavily relies on the high-resolution histology imaging-based cell segmentation and cell type prediction by the histology-based digital (HD)-staining model. The accuracy of HD-staining model should also be directly validated in the several applications described in the paper.*

**Response**: We acknowledge the referee's concern regarding the accuracy of the HD-staining model. To validate the model's ability to accurately identify and classify nuclei, we have rigorously evaluated the results against three diverse cancer datasets through expert analysis. Dr. Liwei Jia, a specialist in pathology and a contributing co-author to this manuscript, has verified the model's reliability.

**Comment 3**: *Since the HD-staining model was trained for cancer cells, it should be clarified what alternatives are to be used for a tissue other than cancer cells without known cell type compositions. If the tool iIMPACT can only handle data related to cancer or data with known cell type compositions, this should be clarified in the abstract and/or paper title.*

**Response**: We value the referee's insights regarding iIMPACT's applicability. We did apply iMPACT to non-cancerous tissues in our analysis of mouse visual cortex STARmap data. To further confirm iIMPACT's applicability in spatial domain identification beyond cancer tissues, we have analyzed the newly included LIBD human dorsolateral prefrontal cortex (DLPFC) data [4], which was obtained using the 10x Visium platform from normal brain tissues. Addressing the challenge of precise cell typing in this context, we have proposed an alternative data preparation pipeline in Figure S13 (attached below), leveraging nuclei segmentation techniques (e.g., Hover-Net [5]) and reference-free cell-type deconvolution methods (e.g., STdeconvolve [9]). The promising findings of this extended analysis are elaborated in Section S3 and visually summarized in Figure S14 (attached below), both available in the supplementary materials. Additionally, in the Discussion section, we have added a paragraph discussing the reliance on nuclei identification methods and the availability of nuclei segmentation methods when high-quality cell typing cannot be achieved (Page 17, Line 1, attached below).

Page 17, Line 1:

*Nuclei identification methods for histology image analysis exhibit several limitations that hinder their widespread applicability and accuracy. One primary challenge is their generalizability. Most deep-learning-based algorithms require model training on high-quality labeled data, making them less adaptable to varied datasets and potentially limiting their generalizability across different tissue types and staining techniques. Besides, the performance of these nuclei identification methods may decrease when handling overlapping nuclei, where segmentation becomes intricate due to the lack of clear boundaries. To address the limitations of existing nuclei identification methods and enhance the versatility of iIMPACT, we proposed an alternative approach for the data preparation outlined in Figure S13 of the supplementary materials. When implementing iIMPACT on tissue sections where precise nuclei classification proves challenging using nuclei identification methods, we leveraged the outputs from deep-learning-based or statistical nuclei segmentation methods to derive the nuclei localization, enabling us to determine the number of nuclei in each spot. Many methods [6-8] exist for isolating cell nuclei across various tissue types without relying on manual labeling data for training. Subsequently, we recommend using reference-free cell-type deconvolution methods [9] to generate the cell type abundance table. This data preparation pipeline was applied to additional SRT data, the LIBD human dorsolateral prefrontal cortex (DLPFC) data generated via 10x Visium [4]. Notably, iIMPACT demonstrated superior performance in spatial domain identification under this alternative approach to generate the image profile (Figure S14 in the supplementary materials). Details are introduced in Section S3 of the supplementary materials. We also validated this data preparation pipeline on the human breast cancer data, and iIMPACT achieved better performance than only utilizing the molecular profile, as shown in Figure S15 in the supplementary materials.*

*Figure S13: An alternative pipeline for data preparation: Reference-free cell type deconvolution algorithms are applied on the molecular profile to generate the cell type abundance table, together with the nuclei localization via nuclei identification methods.*

*Figure S14: LIBD human dorsolateral prefrontal cortex (DLPFC) data: The histology image of the tissue section, the manually annotation provided from the original study, and histology- based spatial domains detected by iIMPACT, SpaGCN, BayesSpace, BASS, stLearn, and MUSE, setting the number of spatial domains to be* $K=7$*.*

**Comment 4**: *As many marker genes are known for various cancer cells, it would improve the utility for users if a quality score of cell type prediction could be provided to the users. For example, the consistency score could be developed to describe the agreement between predicted marker gene expression prediction from histology-based cell type prediction and the gene expression profile from data.*

**Response**: We appreciate the referee's insightful comments. In the revised manuscript, we have utilized two nuclei identification techniques, HD-staining [11] and Hover-Net [5], for advanced nuclei segmentation and classification in histological images. Details of cell type prediction quality are documented in the respective papers; however, this is not the primary focus of our research. To assess the concordance between cell typing from histological imaging and molecular profiling, we applied a recently developed reference-free cell-type deconvolution method, STdeconvolve [9], on the human breast cancer data. The Pearson correlation coefficients of the cell type proportions at spot resolution between the two methods were computed. The comparative analysis, illustrated in the subsequent figure, reveals a high degree of agreement for tumor cells and ductal epithelium while highlighting disparities in some non-tumor cell types like macrophages and stromal cells. This underscores that histology and molecular profiles may align for certain cell types and diverge for others, offering complementary insights across different cell populations.

**Comment 5**: *The choice of the number of domains is briefly mentioned and could be described in more detail. Specifically, which of the examples assumes a prior knowledge of* $K$*? In the examples using a known K, the effectiveness of BIC should be evaluated on its ability to estimate the number of domains.*

**Response**: We are grateful for the referee’s attention to this matter. To clarify our selection process for the optimal $K$, we have included comprehensive guidelines in the Methods section (Page 28, Line 13, attached below). Specifically, we recommend using the integrated completed log-likelihood (ICL) [12] criterion for choosing the best $K$. This criterion's application is demonstrated in Figure S7 (attached below) of the supplementary materials, where the ICL values for various $K$ were computed. Consistently, the ICL minimum at $K=5$ for all three 10x Visium datasets, which was consistent with our pathologist's expert opinion.

Page 28, Line 13:

***Choosing the number of histology-based spatial domains*** $\boldsymbol{K}$*: The number of histology-based spatial domain* $K$*can be determined by prior biological knowledge when available. In the absence of this information, we could apply the integrated completed likelihood (ICL) [12] as the criterion for selecting* $K$*. The ICL is calculated using the following:*

$$\text{ICL}\left( K \right)=-2\log\left( L\left( \boldsymbol{Y,V,}\hat{\boldsymbol{z}} \mid{\hat{\boldsymbol{\mu}}}_{1},\cdots,{\hat{\boldsymbol{\mu}}}_{K}\boldsymbol{,}{\hat{\boldsymbol{\Sigma}}}_{1},\cdots\boldsymbol{,}{\hat{\boldsymbol{\Sigma}}}_{K}\boldsymbol{,}{\hat{\boldsymbol{\omega}}}_{1},\cdots,{\hat{\boldsymbol{\omega}}}_{K} \right) \right)+d\log\left( n \right),$$

*where* $L\left( \boldsymbol{Y,V,}\hat{\boldsymbol{z}} \mid{\hat{\boldsymbol{\mu}}}_{1},\cdots,{\hat{\boldsymbol{\mu}}}_{K}\boldsymbol{,}{\hat{\boldsymbol{\Sigma}}}_{1},\cdots\boldsymbol{,}{\hat{\boldsymbol{\Sigma}}}_{K}\boldsymbol{,}{\hat{\boldsymbol{\omega}}}_{1},\cdots,{\hat{\boldsymbol{\omega}}}_{K} \right)$*is complete data likelihood, i.e., the product of Equation (1) over* $i$*, and* $d=2KP^{'}+K (Q-1)$ *is the total number of model parameters.*

*Figure S7: Integrated completed log-likelihood (ICL) plots for the selection of the number of spatial domains* $K$ *on A. human breast cancer data; B. human prostate cancer data; and C. human ovarian cancer data. The minimum at* $K = 5$ *was selected as the number of spatial domains to analyze for these three SRT data.*

**Comment 6***: For high-resolution ST data like the STARmap example, the data was first coarse-grained and then fed to iIMPACT. Can you directly treat each cell as a spot with a pure cell-type composition and apply iIMPACT without lowering the spatial resolution of the data used?*

**Response**: We thank the referee for this insightful comment. While it is technically feasible to identify spatial domains by treating each cell as a separate spot at the single-cell scale, our results, which we detail below, suggest that this method is less effective than clustering at the spot resolution. One likely explanation is that aggregating single-cell data into grids for spot-level clustering allows for the integration of spatial context from neighboring cells, thereby enhancing the robustness and reliability of the results.

**Comment 7**: *As stLearn is a highly related method, which also uses histology images though in a different way, a comparison to it should be added, for example, clustering results using both gene expression and the morphological features obtained using 'stlearn.pp.extract_feature'.*

**Response**: We thank the referee for highlighting this matter. We have utilized stLearn for spatial clustering on all datasets examined in this study, detailed in Figures 2, 3, 4, and 5 of the main text, as well as Figures S3, S4, S8, S9, S11, S12, S14, and S19 in the supplementary materials. For spatial clustering with stLearn, we employed 'stlearn.pp.extract_feature' to derive morphological features from the histology images, subsequently applying $k$-means clustering to the dimensionality-reduced gene expression data alongside these morphological features.

**Comment 8**: *While using histology-based cell type prediction in domain segmentation for ST data is a novel approach, benefit of this approach needs to be demonstrated. Does HD-staining improve upon using existing cell type deconvolution approaches such as "Miller, Brendan F., et al. "Reference-free cell type deconvolution of multi-cellular pixel-resolution spatially resolved transcriptomics data." Nature Communications 13.1 (2022): 2339."?*

**Response**: We are grateful for the referee’s suggestion. Traditional cell-type deconvolution methods infer cell-type composition at the spot level using molecular profiles and corresponding reference, whereas HD-staining physically identifies cells in histology images. To evaluate the added value of HD-staining to these deconvolution methods, we first used STdeconvolve [9] on the human breast cancer data to determine the proportion of each cell type per spot. Next, we obtained the cell type abundance by multiplying these proportions by the total cell count per spot from HD-staining. Using this cell type abundance data, iIMPACT analysis was performed, and the results are shown in Figure S15 in the supplementary materials (attached below). Incorporating histology-derived cell typing with iIMPACT did yield a higher ARI value.

*Figure S15: Human breast cancer data: The matching histology image of the tissue section with manually annotated labels from pathologists, and spatial domains detected by iIMPACT with nuclei identification by HD-staining on the histology image, iIMPACT with nuclei segmentation by HD-staining on the histology image and cell type deconvolution by STdeconvolve on SRT data, and iIMPACT assuming the image profile is not available, setting the number of spatial domains to be* $K = 5$*.*

**Referee 3: Comments and Responses**

**Comment 1**: *While spatial transcriptomics has enabled us to characterize gene expression while preserving spatial organizing within tissues, current clustering analysis of such gene expression profiles does not utilize histology images to incorporate morphological features alongside molecular information, even though this could improve the accuracy of cluster partitioning. In this manuscript, the authors developed a method, iIMPACT, that uses AI-reconstructed histology images to define histology-based spatial domains (neighboring cells with similar morphological and molecular profiles) through a Bayesian finite mixture model and to identify domain-specific spatially variable genes through a negative binomial regression model. The authors claim that iIMPACT achieved higher accuracy at identifying spatial domains when compared to manually annotated domains by pathologists than existing methods such as BayesSpace and SpaGCN that integrate image features like RGB color values. Among its unique capabilities, the authors claim iIMPACT identifies interaction zones with unique transcriptional profiles. While we found the interaction-zone analyses particularly interesting, we have the following major and minor comments.*

**Response**: We are deeply grateful for the referee's insightful and constructive feedback, as well as the valuable comments provided on our manuscript. We have thoroughly addressed each of the concerns highlighted by the referee in the subsequent sections.

**Comment 2**: *To evaluate performance, the authors compare spatial domains derived from evaluated methods with those derived manually from pathologists. However, pathology annotations are based on only histological information and not molecular information. Any method that integrates both histological and molecular information should presumably identify features that are not perfectly consistent with features derived from just histology. If the authors disregard all molecular information and just use histological information, do they achieve a higher correspondence with pathology annotations? Such a negative control should be included.*

**Response**: We are grateful for the referee's suggestion. Following this suggestion of solely utilizing the image profile derived from HD-staining for iIMPACT, while excluding the molecular and geospatial profiles, we observed a notable decrease in the ARI to only $0.332$ for the human breast cancer dataset, which may be attributed to the high cell type mixture across domains, presenting a complexity in clustering analysis. In contrast, an iIMPACT analysis that integrates all three profiles yielded a higher ARI of $0.634$, as depicted in Figure 2B of the main text. Such findings underscore the criticality of integrating molecular information.

**Comment 3***: Given that many of these methods are sensitive to parameters, what if the number of domains was decreased? Could the accuracy increase? In general, sensitivity to parameters must be evaluated.*

**Response**: We appreciate the referee pointing out this issue. To address this point, we have added Figure S4 in the supplementary materials (attached below), which presents the accuracy of spatial domain identification across varying numbers of spatial domains $K$, indicating that a reduction in $K$ does not necessarily correlate with an improved ARI for most methods. Furthermore, Figures S3, S9, and S12 have been added to illustrate the spatial domain patterns generated by different methods when varying the prespecified $K$ for human breast, prostate, and ovarian cancer data, respectively.

*Figure S4: ARIs achieved by six spatial domain identification methods, iIMPACT, SpaGCN, BayesSpace, BASS, stLearn, and MUSE, under different choices of the number of spatial domains*$K$ *on the A. human breast cancer data; B. human prostate cancer data; and C. human ovarian cancer data.*

**Comment 4:** *More conceptually, what if the tumor region does have two subpopulations that are molecular distinct or even morphologically but the pathologists were not asked to differentiate this when making their manual annotations? More broadly, should pathologist annotations of histology images be used as ground truth for methods that incorporate molecular information to do classification? A discussion of this is warranted.*

**Response**: We are thankful for the referee's attention to this matter. We recognize that manual annotations by pathologists may not represent the "true" segmentation of regions based on an integration of morphological and molecular data. Nonetheless, it remains a standard practice within the field of spatial domain identification to use manual annotation as a benchmark, as evidenced by its widespread application in foundational works such as BayesSpace [14], SpaGCN [13], BASS [1]. We also acknowledge that the suitability of manual annotations as a proxy for ground truth has not been extensively challenged in literature focused on spatial domain identification, a discussion that often extends beyond the ambit of such methodology-centric publications. In light of these considerations, we have avoided using the term "ground truth" and replaced the term with "manual annotation" throughout the manuscript.

**Comment 5**: *Likewise, the authors quantified performance based on adjusted rand index for evaluating consistency between spatial domains and manual annotations. While iIMPACT appears to have done better (ARI* $=0.634$*) compared to other methods (ARI*$=0.520$ *for SpaGCN and ARI* $=0.419$ *for BayeSpace), these seem all to be quite poor performance. What is a baseline level of performance and does a* $0.1$ *ARI indicate a substantial and significant improvement?*

**Response**: We understand the referee's concern regarding the significance level of ARI, a standard metric for assessing clustering performance. To provide a visual representation of how ARI values correlate with clustering performance, we have prepared an explanatory figure, Figure S2 in the supplementary materials (attached below). We hope this additional figure enhances readers' understanding of this key summary statistic and its practical implications.

*Figure S2: A graphical illustration of the adjusted Rand index (ARI).*

**Comment 6**: *In Figures 2F, 3F, and 4F, SPARK has* $0$ *overlap. Is this because SPARK failed to run? Or because it really gave results that had no overlap?*

**Response**: We successfully applied SPARK to the human breast, prostate, and ovarian cancer 10x Visium data, and identified a number of SVGs, using a threshold of $0.05$ for FDR-corrected $p$-values. The identified SVGs, however, did not align with known genes from established lists, likely due to the intrinsic complexity of the 10x Visium data. While a relaxation of the threshold produced an increased number of SVGs, we opted against this approach to preserve the robustness and scientific rigor of our findings.

**Comment 7**: *In the breast cancer data evaluated, fat tissue doesn't seem to be represented in any of the domains identified by iIMPACT or other methods (Page 8, Line 27). Does that mean you would not expect to recapitulate a fat spatial domain? This limitation should be discussed along with any other cell-type or domain-specific limitations.*

**Response**: We are grateful to the referee for highlighting this issue. We acknowledge that every method has its constraints in spatial domain identification, particularly when it comes to segregating non-tumor regions, such as differentiating fat tissue as shown in Figure 2A. In response, we have added a discussion about it.

Page 8, Line 20:

*Notably, none of the methods performed well in separating the fat region (in blue) from the fibrous tissue (in red) per the manual annotation.*

**Comment 8**: *We were unclear as to what is the advantage of using cell types from HD-staining to interpret domains instead of using molecular information?*

**Response**: We thank the referee for raising this great question. Numerous methods for spatial domain identification necessitate a dimensionality reduction step applied to the molecular profile, which compromises the clarity and direct interpretability of the identified spatial domains. On one hand, the cell type abundance data derived from HD-staining encompasses comprehensive morphological details, such as cell growth pattern, cell-cell interaction, and cell interaction with the surrounding microenvironment, thereby improving the correlation between the identified spatial domain patterns and the manual annotations commonly employed as benchmarks. On the other side, the latent spatial domain-specific relative abundance of cell types parametrized by iIMPACT offers a straightforward and user-friendly approach to define and characterize the identified spatial domains.

**Comment 9**: *The authors suggest that "identified zones have a high level of heterogeneity in gene expression and complex cellular interactions" seems like an excessive claim (Page 9, Line 42). We were unsure whether the level of heterogeneity that exists in these interaction zones was any more significant than the result of profiling spots that have a mix of two cell types. The authors need to be able to differentiate if this "gene expression richness" is not just the result of mixing two cell types and instead the result of complex cellular interactions to support their claim.*

**Response**: We thank the referee for highlighting this issue. The metric of "gene expression richness" presented in Figures 2E, 3E, and 4E is calculated for each spot within the boundaries of the tumor region, the boundaries of the immune region, and the interactive zone, rather than at the single-cell level across various cell types. Our designation of interactive zone—areas with greater uncertainty in spatial domain allocation—is to emphasize their gene expression diversity, which was notably higher compared to other regions we have characterized.

**Comment 10**: *The analysis of domain-specific SVGs here seems to confound ideas of SVGs (genes with spatial autocorrelation) with differentially expressed genes (DEGs) between domain. In the methods, the authors define SVGs as "differentially expressed in a given histology-based spatial domain compared with all other domains." (Page 27, Line 26) The authors do not describe how they used SpaGCN, SpatialDE, and SPARK to get SVGs so it is not clear if these are actually SVGs or also DEGs. We suggest the authors replace uses of SVG with DEG between domains where appropriate.*

**Response**: We agree with the referee's opinion on the difference between DEGs and SVGs. Our study focuses on identifying genes that show differential expression within specific histology-based spatial domains as opposed to other domains. Accordingly, we propose to refer to these genes as "spaDEGs," an abbreviation for spatial domain-specific differentially expressed genes. We have updated the term "SVG" to "spaDEG" throughout our manuscript to reflect this more accurate description.

**Comment 11**: *Similar to ARI, we had a difficult time interpreting the fold enrichment (odds ratio) graphs and how it showed that "compared with other SVG detection methods, iIMPACT-defined SVGs are more enriched of known functional genes" and support the claim that "iIMPACT could provide a better understanding of both cellular spatial organization and functional gene landscape of developmental and diseased tissues." (Page 15, Line 39). It is not clear to us what a baseline level of performance would be and whether iIMPACT's* $1.7\times{10}^{-14}$ *represents a substantial and significant improvement over SpaGCN at* $8.0\times{10}^{-10}$ *for example.*

**Response**: In this analysis, we compare the identified spaDEGs identified by iIMPACT and SVGs detected by other methods with a well-established list of cancer-related genes. The fold enrichment, which represents the odds ratio in comparison to random overlap, is plotted on the y-axis of Figures 2F, 3F, 4F, and 5F. The $p$-values displayed in these figures indicate the statistical significance of the observed fold enrichments. For greater clarity, we have revised the figures to directly illustrate the fold enrichment values attained by each method.

**Comment 12**: *Overall, it is not clear the sensitivity of iIMPACT as an analysis method. The authors claim that iIMPACT identifies genes that are interpretable. But how many of these are false positives? For example, the authors analyzed 1020 genes in STARmap (Page 13) and described two iIMPACT-defined SVGs as being functionally relevant. How many genes did iIMPACT identify? Just these two?*

**Response**: We are thankful to the referee for this observation. Quantifying the false positives among the identified spaDEGs presents a challenge in our study due to the absence of experimentally validated ground truths for comparison. Accordingly, we have revised our description as "iIMPACT is an interpretable method" with respect to spaDEG detection.

**Comment 13**: *The authors describe spatially resolved transcriptomics as a "new generation of RNA sequencing analysis techniques". We find this description to mischaracterize imaging-based spatial transcriptomics technologies, which are a new generation of smFISH not sequencing. We believe "new generation of RNA profiling techniques" would be more appropriate.*

**Response**: We are grateful to the referee for drawing attention to this detail. In line with this suggestion, we have revised the term from "RNA sequencing analysis techniques" to "RNA-profiling techniques" to enhance the precision of our statement.

Page 3, Line 2:

*Spatially resolved transcriptomics (SRT), a new generation of RNA profiling techniques, provides biological information at the cellular level while preserving the organization of the tissue and cellular microenvironment.*

**Comment 14**: *The authors claim that image features from stLearn, hidden-Markov random field, BayesSpace, and SpaGCN "fail to provide biologically relevant insight" (Page 4, Line 12). We believe this is an overstatement and very dismissive of prior work that has already been used to by others to derive biological insights.*

**Response**: We regret any previous overstatement and have revised this sentence (Page 4, Line 7, attached below) accordingly.

Page 4, Line 7

*However, those features do not explicitly reveal detailed morphological information (e.g., cell locations and types) and, thus, have limited ability to directly provide biologically or clinically relevant insights.*

**Comment 15**: *The authors claim that their tool is uniquely able to "interpret the identified domains in a biologically meaningful way" (Page 9, Line 4), but this is something that people usually do downstream of clustering into domains or cell type by using tools for differential gene expression analysis.*

**Response**: We recognize that performing downstream analyses could provide additional biological insights after spatial domain clustering. However, the novelty of iIMPACT is its integrated approach, which enables the identification of spatial domains, while estimating their respective cell type relative abundances, allowing for immediate interpretation. This joint modeling approach typically outperforms multi-stage methods, which can yield sub-optimal results. We have made this advantage more explicit in the revised main text, specifically on Page 9, Line 3.

Page 9, Line 3:

*Secondly, iIMPACT is able to define each individual histology-based spatial domain simultaneously by inferring the latent spatial domain-specific relative abundance of cell types parametrized by the Bayesian multinomial-normal mixture model (Figure 2C). In contrast, other methods, despite their good capabilities in identifying spatial domains, currently lack the ability to effectively integrate cell type information and directly interpret the identified domains in a biologically meaningful way.*

**Comment 16**: *We did not find the genes visualized in Figure 5D to be as compelling as SVGs. Perhaps an alternative visualization would be better.*

**Response**: We appreciate the referee's attention to this matter. We acknowledge that the spatial expression patterns of the two genes uniquely identified by iIMPACT may not be immediately discernible, despite their statistical significance and biological relevance. To enhance clarity, we have added bar plots in Figure 5D (attached below). It depicts a significantly higher proportion of non-zero expression in the given histology-based spatial domain than others, which might contribute to the differential expression.

**Comment 17**: *The paper would benefit from explicitly defining AI-reconstructed (Page 4, Line 41).*

**Response**: We are thankful to the referee for this suggestion, which has enhanced the rigor of our manuscript. In response, we have explicitly defined the term "AI-reconstructed histology image" within the text to ensure precise comprehension for our readers.

Page 4, Line 17:

*Thus, we hypothesize that integrating spot-level molecular profiles and cellular-level image profiles from AI-reconstructed histology images—digitally processed tissue samples with AI-identified and classified nuclei, primarily using deep learning—could enhance the spatial domain identification in terms of both accuracy and interpretability.*

**References**

1.	Li Z, Zhou X: **BASS: multi-scale and multi-sample analysis enables accurate cell type clustering and spatial domain detection in spatial transcriptomic studies.** *Genome Biology* 2022, **23:**168.

2.	Bao F, Deng Y, Wan S, Shen SQ, Wang B, Dai Q, Altschuler SJ, Wu LF: **Integrative spatial analysis of cell morphologies and transcriptional states with MUSE.** *Nature Biotechnology* 2022, **40:**1200-1209.

3.	Pham D, Tan X, Xu J, Grice LF, Lam PY, Raghubar A, Vukovic J, Ruitenberg MJ, Nguyen Q: **stLearn: integrating spatial location, tissue morphology and gene expression to find cell types, cell-cell interactions and spatial trajectories within undissociated tissues.** *bioRxiv* 2020**:**2020.2005.2031.125658.

4.	Maynard KR, Collado-Torres L, Weber LM, Uytingco C, Barry BK, Williams SR, Catallini JL, Tran MN, Besich Z, Tippani M: **Transcriptome-scale spatial gene expression in the human dorsolateral prefrontal cortex.** *Nature Neuroscience* 2021, **24:**425-436.

5.	Graham S, Vu QD, Raza SEA, Azam A, Tsang YW, Kwak JT, Rajpoot N: **Hover-Net: Simultaneous segmentation and classification of nuclei in multi-tissue histology images.** *Medical Image Analysis* 2019, **58:**101563.

6.	Hayakawa T, Prasath VS, Kawanaka H, Aronow BJ, Tsuruoka S: **Computational nuclei segmentation methods in digital pathology: a survey.** *Archives of Computational Methods in Engineering* 2021, **28:**1-13.

7.	Mouroutis T, Roberts SJ, Bharath AA: **Robust cell nuclei segmentation using statistical modelling.** *Bioimaging* 1998, **6:**79-91.

8.	Fatakdawala H, Xu J, Basavanhally A, Bhanot G, Ganesan S, Feldman M, Tomaszewski JE, Madabhushi A: **Expectation–maximization-driven geodesic active contour with overlap resolution (emagacor): Application to lymphocyte segmentation on breast cancer histopathology.** *IEEE Transactions on Biomedical Engineering* 2010, **57:**1676-1689.

9.	Miller BF, Huang F, Atta L, Sahoo A, Fan J: **Reference-free cell type deconvolution of multi-cellular pixel-resolution spatially resolved transcriptomics data.** *Nature Communications* 2022, **13:**2339.

10.	Li H, Calder CA, Cressie N: **Beyond Moran's I: testing for spatial dependence based on the spatial autoregressive model.** *Geographical Analysis* 2007, **39:**357-375.

11.	Wang S, Rong R, Yang DM, Fujimoto J, Yan S, Cai L, Yang L, Luo D, Behrens C, Parra ER, et al: **Computational staining of pathology images to study the tumor microenvironment in lung cancer.** *Cancer Research* 2020, **80:**2056-2066.

12.	Biernacki C, Celeux G, Govaert G: **Assessing a mixture model for clustering with the integrated completed likelihood.** *IEEE transactions on Pattern Analysis and Machine Intelligence* 2000, **22:**719-725.

13.	Hu J, Li X, Coleman K, Schroeder A, Ma N, Irwin DJ, Lee EB, Shinohara RT, Li M: **SpaGCN: Integrating gene expression, spatial location and histology to identify spatial domains and spatially variable genes by graph convolutional network.** *Nature Methods* 2021, **18:**1342-1351.

14.	Zhao E, Stone MR, Ren X, Guenthoer J, Smythe KS, Pulliam T, Williams SR, Uytingco CR, Taylor SEB, Nghiem P, et al: **Spatial transcriptomics at subspot resolution with BayesSpace.** *Nature Biotechnology* 2021, **39:**1375-1384.

**Second round of review**

**Reviewer #1:**

This revised version has seen improvements with additional evaluations compared to the original one. However, there are still key concerns that remain inadequately addressed:

1. The comparative analyses in Figure S4 show that iIMPACT offers minimal improvement in the breast cancer dataset compared to BASS, and it is consistently outperformed by both BASS and BayesSpace in the prostate cancer dataset. In the ovarian cancer dataset, iIMPACT's higher ARI over BayesSpace is apparent only when the number of clusters equals to 2, which is not a typical practice in the field. Overall, the advancements of iIMPACT appear to be incremental in the spatial clustering task, particularly compared to BayesSpace and BASS.

2. Regarding my previous concern about iIMPACT's reliance on accurate cell typing from HD-staining, the authors suggest alternatives like Hover-Net and STdeconvolve. However, HD-staining is tailored for lung cancer, and Hover-Net for colorectal adenocarcinoma, limiting their applicability. In other tissues, iIMPACT uses STdeconvolve for cell type deconvolution, leading to inconsistency in analyses across different tissue types and potentially biased results.
Additionally, the use of STdeconvolve makes iIMPACT quite similar to BASS, as both methods incorporate cell type composition in their spatial clustering. Given BASS's recent publication in Genome Biology, such methodological overlap raises questions about iIMPACT's uniqueness and contribution to the field.

3. iIMPACT presents practical disadvantages compared to other methods.
1), iIMPACT's analysis pipeline is not consistent across different tissue types, while other methods of the same goal including BASS, BayesSpace, and SpaGCN, can analyze ST data from various tissue types, both with/without H&E images.
2) The authors claim that one difference between iIMPACT and BASS is that BASS supports multi-sample clustering. As the field of ST is moving towards multi-sample studies, I consider this a significant advantage of BASS over iIMPACT. Considering the high methodological similarities between iIMPACT and BASS, I would insist that iIMPACT is not superior to BASS and adds limited value to this field.

4. My concerns extend to iIMPACT's contribution to identify interactive regions. While the authors claim this as a unique feature, Figure S3 shows that other methods, including SpaGCN, BayesSpace, and BASS, can also identify these regions. Notably, SpaGCN achieves more consistent results across different clustering levels (K values) than iIMPACT. As all these methods offer prediction uncertainty through their soft clustering approach, iIMPACT's ability to quantify uncertainty in defining interactive regions does not stand out as a major improvement.

**Reviewer #2:**

The authors have addressed all my concerns and I believe the manuscript has been substantially improved.

**Reviewer #3:**

We thank the authors for their thorough and well organized review as well as the other reviewers for bringing our attention to matters we had missed. The authors have addressed a majority of our original concerns. In particular, we appreciate the new comparison with other spatial domain detection methods across a range of parameters and datasets. However, many of our concerns, while addressed in the point-by-point response, did not appear incorporated into the actual manuscript. We believe the manuscript would be improved if the responses were better incorporated into the manuscript.

Specifically, we have the following outstanding concerns:

- Regarding Response to Referee3-Comment2: We appreciate the new analysis demonstrating that excluding the molecular and geospatial profiles, ARI was decreased to only 0.332. The authors attribute this to the high cell-type mixture across domains, which should be visualized. We believe this result highlights the utility of iIMPACT's integration of molecular and imaging profiles to yield a comparatively higher ARI of 0.634. The result of decreased ARI of 0.332 when imaging profiles are not used along with the high cell-type mixtures across domains should be incorporated into the actual manuscript.

- Regarding Response to Referee3-Comment4: We agree with the authors that while the manual annotations by pathologists may not represent the true segmentation of regions, it remains standard practice in a machine-learning framework. However, this is still a limitation that warrants acknowledgement in the discussion.

- Regarding Response to Referee3-Comment7: We appreciate the authors noting that none of the methods performed well in separating the fat region from the fibrous tissue. However, our concerns were more broad. Fat (and perhaps also other tissue domains) and histologically distinct but molecular difficult to characterize and, as such, integration methods that rely on molecular information may not adequately capture such tissue domains. A discussion of why none of the methods performed well in separating the fat region from the fibrous tissue is warranted.

- Regarding Response to Referee3-Comment8: We appreciate the author's clarification that "cell type abundance data derived from HD-staining encompasses comprehensive morphological details..." While "latent spatial domain-specific relative abundances of cell types parametrized by iIMPACT offers [an] approach to define and characterize identified spatial domains." We believe this text should be included in the manuscript and not just as a response to reviewer.

- Regarding Referee3-Comment9: We found the author's clarification of the description of gene expression richness provided in the reviewer response to be helpful in our understanding of the concept. We believe this text should be included in the manuscript and not just as a response to reviewer.

- Regarding Response to Referee3-Comment12: We agree with the authors that there is a lack of experimentally validated ground truths for estimating false positives. However, we still find it is important to explicitly clarify the number of genes identified by iIMPACT. For example, a reader's interpretation of the result could be different if iIMPACT identified 10/1020 with 2 being known true positives or if it identify 1000/1020 with 2 being known as true positives. We found this information difficult to discern from the supplementary figures alone.

**Authors Response**

**Point-by-point responses to the reviewers’ comments:**

**Chief Editor: Comments and Responses**

**Comment 1:** *In particular, it seems to us to be essential that you address Referee 3's concerns about implementing the responses to the reviewers in the manuscript text.*

**Response:** We acknowledge the oversight of not including responses to Referee 3 in our previous manuscript text. This decision was made in an effort to maintain focus and comply with page limitations. Recognizing the value of incorporating these responses, we have now integrated these responses into the latest revised manuscript text. For further details, please refer to our expanded responses to Referee 3 in this letter.

**Comment 2:** *We also request that you analyse the reason for lack of consistent performance mentioned by Reviewer #1 in point 3. When consulted on this, Reviewer #3 believes that the performance is better on those samples where the transcriptional information was more noisy or perhaps sparse due to sample quality issues and therefore histological information became more informative. They anticipate this may explain the performance variation across evaluated tissues. However, this should be demonstrated in practice.*

**Response:** To maintain methodological consistency across our analyses, we have employed the iIMPACT framework with the two data preprocessing pipelines, as proposed in Figure 1 and Figure S13, for all three cancer datasets analyzed in our study. The details are provided in our responses to Referee 1’s Comment 3 and Referee 2’s Comment 2 in this letter. The results visualized in the updated Figure S15, as well as in the newly included Figures S16 and S17, illustrate that iIMPACT, when utilizing only HD-Staining to generate image profiles, consistently surpassed most of the methods in most of the scenarios, achieving ARI scores of $0.634$, $0.674$, and $0.967$ for breast, prostate, and ovarian cancer datasets, respectively. Notably, depending solely on the image profile led to a significant decline in ARI values due to the high cell-type mixture across spatial domains, as visualized in the newly added Figure S18, whereas iIMPACT's use of only the molecular profile yielded satisfactory results, with ARI scores of $0.494$, $0.668$, and $0.808$, respectively.

We acknowledge Referee 3's hypothesis that iIMPACT's performance may correlate with the quality of the molecular profile, such as the sparsity of the gene expression count table. This hypothesis is reasonable. However, testing this hypothesis may require a large amount of spatial transcriptomic datasets, and is beyond the score of this paper. Furthermore, testing this hypothesis is challenging due to the multitude of influencing factors, and the validity of using sparsity as a quality metric is debatable. For instance, the sparsity levels for the three cancer datasets are $73\%$, $74\%$, and $61\%$, respectively, yet we observed no distinct correlation between ARI scores and sparsity levels. This could be attributed to iIMPACT's preprocessing approach, which selects the top $2,000$ highly variable genes and applies dimensionality reduction through PCA.

**Comment 3:** *Related to Comment 4 by Reviewer #1, the authors should indeed clarify that the interaction regions identified by iIMPACT were also identified as distinct spatial domains by other methods. However, Reviewer #3 finds iIMPACT's designation of these domains as interaction regions rather than just another spatial domain useful in its interpretation. Therefore, Reviewer #3 requests that you clarify the description of the interaction region and definition of gene expression richness may assist in clarifying this point.*

**Response:** We express our gratitude to the editor for highlighting this shared concern raised by Referees 1 and 3. For further details, please refer to our responses to Referee 1's Comment 5 and Referee 2's Comment 6 in this letter. In brief, the interactive zones identified by iIMPACT are conceptually distinct from the mutually exclusive spatial domains identified by other methods. This distinction arises because iIMPACT employs the variability inherent in the spatial domain allocations, specifically second-order summary statistics, as opposed to defining spatial domains based on their most probable allocations, or first-order summary statistics. Our real data analysis has further demonstrated that these interactive zones are characterized by a notably higher gene expression richness. In accordance with Referee 2's suggestion, we have provided a more explicit definition of these interactive zones in our revised manuscript text.

**Comment 4:** *We are considering your manuscript as a 'Method' article. This is our format for publishing articles that describe a methodological innovation that is a significant advance over published methods and likely to be of broad utility, but that do not provide significant biological insights. When revising the manuscript, please ensure the manuscript conforms to our style for Methods articles.*

**Response:** We are grateful for the editor's recommendation. We have ensured our revision to conform to the stylistic guidelines of a 'Method' article.

**Referee 1: Comments and Responses**

**Comment 1**: *This revised version has seen improvements with additional evaluations compared to the original one. However, there are still key concerns that remain inadequately addressed.*

**Response**: We are thankful for the referee's recognition of the improvements in our initial revision. We are committed to thoroughly addressing the remaining concerns in our detailed responses below.

**Comment 2**: *The comparative analyses in Figure S4 show that iIMPACT offers minimal improvement in the breast cancer dataset compared to BASS, and it is consistently outperformed by both BASS and BayesSpace in the prostate cancer dataset. In the ovarian cancer dataset, iIMPACT's higher ARI over BayesSpace is apparent only when the number of clusters equals to 2, which is not a typical practice in the field. Overall, the advancements of iIMPACT appear to be incremental in the spatial clustering task, particularly compared to BayesSpace and BASS.*

**Response**: We respectfully disagree with the referee's assessment regarding this crucial point. Figure S4 was designed to thoroughly assess the performance of various methods in identifying spatial domains, especially when the number of spatial domains $K$ is misspecified. To ensure a fair comparison, it is essential to select a justifiable value of $K$; failing to do so could lead to a cherry-picking issue, undermining the validity of the comparison. Therefore, referencing Figure S4, we have made an additional table (Table S4, attached below) to offer a more practical comparison of clustering performance between iIMPACT, BayesSpace and BASS where $K$ was determined by either experienced pathologists or the integrated completed likelihood (ICL) criterion. The results decisively indicate that iIMPACT outperforms BayesSpace and BASS in five out of six scenarios. Notably, these are not marginal improvements. When $K$ was selected by pathologists, the average ARI improvements were $34\%$ over BayesSpace and $17\%$ over BASS. When $K$ was derived from the ICL, the improvements were $13\%$ and $20\%$ over BayesSpace and BASS, respectively. The notable improvement in performance is credited to iIMPACT's effective use of information from corresponding histological images, which is extensively validated in Figure S15 (for the human breast cancer dataset), and further supported by the newly added Figures S16 and S17 (for the human prostate and ovarian cancer datasets, respectively). In these figures, we have quantified the contributions of molecular and imaging information, illustrating how they collectively lead to a higher ARI.

Furthermore, iIMPACT distinctively enables a clear interpretation of spatial domains through domain-level cell type abundance, a critical component for the analysis of NGS-based SRT datasets. While BASS shares a similar capability for NGS-based SRT data when treating each spot as a “pseudo cell,” it no longer has the cell type interpretation, as clearly claimed by the BASS paper. iIMPACT's unique feature not only differentiates it from BASS but also equips biologists with valuable insights for subsequent domain analysis.

**Comment 3**: *Regarding my previous concern about iIMPACT's reliance on accurate cell typing from HD-Staining, the authors suggest alternatives like Hover-Net and STdeconvolve. However, HD-Staining is tailored for lung cancer, and Hover-Net for colorectal adenocarcinoma, limiting their applicability. In other tissues, iIMPACT uses STdeconvolve for cell-type deconvolution, leading to inconsistency in analyses across different tissue types and potentially biased results. Additionally, the use of STdeconvolve makes iIMPACT quite similar to BASS, as both methods incorporate cell-type composition in their spatial clustering. Given BASS's recent publication in Genome Biology, such methodological overlap raises questions about iIMPACT's uniqueness and contribution to the field.*

**Response**: We value the referee's insights on the data preprocessing step for generating image profiles for iIMPACT. To address the concern about the iIMPACT’s dependence on precise cell-typing from HD-Staining, we validated that iIMPACT's performance was robust between the two imaging data preprocessing strategies (outlined in Figure 1 and Figure S13), as indicated by the results with human breast cancer data in Figure S15. To ensure consistency and address the referee's concern comprehensively, we have applied iIMPACT using these two data preprocessing strategies to the human prostate and ovarian cancer datasets. The outcomes, illustrated in the newly added Figures S16 and S17, are also summarized in Table S5 (attached below). Although HD-Staining was designed for lung cancer, our iIMPACT with HD-Staining consistently outperformed BASS. Moreover, using HD-Staining for nuclei segmentation alongside STdeconvolve for classification proved to be competitive with BASS in identifying spatial domains. We have further addressed this point by updating our data preparation pipeline recommendations for various tissue types in the revised Conclusion and Discussion section (see Page 19, Lines 2-9). For ease of reference, we have copied the new discussion and Figures S16 and S17 below.

In addressing the referee’s concerns regarding methodological similarities between BASS and iIMPACT, it is critical to note that iIMPACT utilizes cell-type composition data from HD-Staining or STdeconvolve as observed inputs. This contrasts with BASS, which needs to pre-specify the number of cell types and estimate cell-type composition within its model. This key difference in modeling strategy clearly differentiates iIMPACT from BASS; let alone iIMPACT and BASS were designed for analyzing NGS-based and imaging-based SRT data, respectively. Considering iIMPACT's incorporation of additional cell type relative abundance data, we expect it to outperform BASS, especially when molecular and histological data are less optimally aligned.

On Page 19:

*…To leverage the histology image more sufficiently and enhance the interpretability of identified domains, we suggest obtaining the image profile, i.e., the cell type abundance table, by conducting nuclei identification through HD-Staining for cancer tissues. While originally designed for lung cancer, HD-Staining has proven effective for breast, prostate, and ovarian cancers, as demonstrated in our study, indicating its broader utility. The alternate data preparation pipeline should be reserved for instances where HD-Staining is less effective, such as with non-cancerous tissue sections…*

**Comment 4**: *iIMPACT presents practical disadvantages compared to other methods.*

*1) iIMPACT's analysis pipeline is not consistent across different tissue types, while other methods of the same goal including BASS, BayesSpace, and SpaGCN, can analyze ST data from various tissue types, both with/without H&E images.*

*2) The authors claim that one difference between iIMPACT and BASS is that BASS supports multi-sample clustering. As the field of ST is moving towards multi-sample studies, I consider this a significant advantage of BASS over iIMPACT. Considering the high methodological similarities between iIMPACT and BASS, I would insist that iIMPACT is not superior to BASS and adds limited value to this field.*

**Response**:

Regarding 1), We apologize for any confusion regarding consistency in our previous revision. To guarantee consistent data preprocessing, we have implemented the two data preprocessing strategies (as depicted in Figure 1 and Figure S13) across all three human cancer datasets. Importantly, iIMPACT can function without H&E images, similar to other methods like BASS, BayesSpace, and SpaGCN. Uniquely, iIMPACT also functions without gene expression data, which is not possible with other methods. However, disregarding available information might compromise the results. We have summarized the above results, which favor iIMPACT in Figures S15, S16, and S17 (attached in our response to Referee 1’s Comment 3). The significant enhancement in performance should be attributed to iIMPACT's effective utilization of information from corresponding histological images, a common practice in all NGS-based SRT experiments. For non-cancerous tissues where H&E staining is inapplicable, iIMPACT accommodates an alternative approach using Hover-Net for nuclei segmentation and STDeconvolve for nuclei classification, which also produces satisfactory results in the LIBD human dorsolateral prefrontal cortex (DLPFC) data (see Figure S14).

Regarding 2), please refer to our response to Referee 1’s Comment 3 for the distinct differences between BASS and iIMPACT in the previous response letter, along with our reply to Comment 3 in this letter. We agree with the referee that the field of spatial domain identification is progressing towards multi-sample analyses. Unlike BASS, which is dependent solely on molecular data, iIMPACT leverages both molecular and histological imaging data. This integration of histology images necessitates aligning multiple tissue sections before multi-sample analysis, a process beyond this paper's scope. Despite the extra steps required to create image profiles, we assert that the inclusion of histological data is crucial for enhancing spatial clustering, as evidenced by our single-sample study results (e.g., see Figures S15, 16, and S17).

**Comment 5**: *My concerns extend to iIMPACT's contribution to identify interactive regions. While the authors claim this as a unique feature, Figure S3 shows that other methods, including SpaGCN, BayesSpace, and BASS, can also identify these regions. Notably, SpaGCN achieves more consistent results across different clustering levels (K values) than iIMPACT. As all these methods offer prediction uncertainty through their soft clustering approach, iIMPACT's ability to quantify uncertainty in defining interactive regions does not stand out as a major improvement.*

**Response**: We acknowledge the referee's concerns regarding the identification of interactive regions, a distinct aspect of iIMPACT. While soft clustering methods like SpaGCN, BayesSpace, and BASS provide uncertainty in domain allocation, our approach is pioneering in quantitatively defining interactive regions through posterior probabilities and uncovering those interactive regions' features, including a higher diversity in cell type abundance and gene expression heterogeneity (see Figure 2E, 3E, and 4E). Moreover, obtaining domain allocation uncertainty is not straightforward with SpaGCN, BayesSpace, and BASS in practice. In contrast, our software provides a function named get.interactive.zone() to return those interactive regions immediately.

In response to the remarks in Figure S3 that “*SpaGCN achieves more consistent results across different clustering levels (*$K$ *values) than iIMPACT*,” we hold a different view. While we recognize the merits of SpaGCN, our iIMPACT consistently achieved higher ARIs in the referenced human breast cancer dataset, as evidenced by the subsequent Figure S4A. The strategy of expanding the spatial domain $K$ to identify interactive regions warrants further discussion because it can yield suboptimal outcomes, as we have to assume the interactive regions and spatial domains are mutually exclusive. However, iIMPACT naturally relaxes this constraint, using the first-order information from the posterior probability on domain allocation to determine the spatial domain and the second-order information to define the interactive regions.

**Referee 3: Comments and Responses**

**Comment 1**: *We thank the authors for their thorough and well-organized review as well as the other reviewers for bringing our attention to matters we had missed. The authors have addressed a majority of our original concerns. In particular, we appreciate the new comparison with other spatial domain detection methods across a range of parameters and datasets. However, many of our concerns, while addressed in the point-by-point response, did not appear incorporated into the actual manuscript. We believe the manuscript would be improved if the responses were better incorporated into the manuscript.*

**Response**: We sincerely appreciate the referee's constructive feedback. Our apologies for omitting responses in the previous manuscript text; this was to maintain focus and adhere to page constraints. We agree that the inclusion of those responses would further improve the paper. Please find the detailed amendments prompted by this comment below.

**Comment 2**: *Regarding Response to Referee3-Comment2: We appreciate the new analysis demonstrating that excluding the molecular and geospatial profiles, ARI was decreased to only 0.332. The authors attribute this to the high cell-type mixture across domains, which should be visualized. We believe this result highlights the utility of iIMPACT's integration of molecular and imaging profiles to yield a comparatively higher ARI of 0.634. The result of decreased ARI of 0.332 when imaging profiles are not used along with the high cell-type mixtures across domains should be incorporated into the actual manuscript.*

**Response**: We thank the referee for this valuable suggestion. To evaluate the contributions of histological and molecular information to the Adjusted Rand Index (ARI), we have applied iIMPACT to three human cancer datasets under three different scenarios: 1) utilizing both molecular and histological image profiles, 2) using only the molecular profile, and 3) using only the histological image profile. The comparative results are illustrated in Figure S15, as well as in the newly added Figures S16 and S17, corresponding to the human breast, prostate, and ovarian cancer datasets, respectively. Additionally, we have prepared a table below to summarize the performance across these three scenarios. It was observed that relying solely on the image profile resulted in a significant reduction in ARI values, whereas iIMPACT's use of only the molecular profile yielded satisfactory results.

|  | Number of domains $\boldsymbol{K}$ | Adjusted Rand Index (ARI) | | |
| --- | --- | --- | --- | --- |
|  |  | iIMPACT w/ both profiles | iIMPACT w/ molecular profile | iIMPACT w/ image profile |
| Human breast cancer dataset | 5 | **0.634** | 0.494 | 0.332 |
| Human prostate cancer dataset | 3 | **0.674** | 0.668 | 0.312 |
| Human ovarian cancer dataset | 2 | **0.967** | 0.808 | 0.102 |

We apologize for not providing sufficient detail previously when discussing the low ARIs associated with using only the histological image profile, which we attributed to the high cell-type mixture across domains. To address this, we have created Figure S18, which presents the cell-type mixture from three angles: 1) cell-type composition across spots, 2) the number of cell types across spot, and 3) the Shannon equitability index [1] based on relative cell type abundance across spots. Figure S18A reveals a discernible spatial pattern in the cell-type composition across spots, indicating that spatial clustering may be viable when employing histological information only. Notably, over $70\%$ of spots contain four or more cell types, and the Shannon equitability index confirms the extensive cell-type mixture within spots and across domains. We have expanded on these insights in the Conclusion and Discussion section (refer to Page 19, Lines 16-20). For ease of reference, the related discussion and Figures S15, S16, and S17 are copied below.

On Page 19:

*…Third, iIMPACT's performance in spatial domain identification was less satisfactory when dependent solely on histological image profiles, as shown in Figures S15-17. This may be due to the extensive cell-type heterogeneity within domains, exemplified in Figure S18 for the human breast cancer dataset. Thus, integrating molecular information is crucial for effective spatial clustering…*

**Comment 3**: *Regarding Response to Referee3-Comment4: We agree with the authors that while the manual annotations by pathologists may not represent the true segmentation of regions, it remains standard practice in a machine-learning framework. However, this is still a limitation that warrants acknowledgement in the discussion.*

**Response**: We are thankful for the referee's attention to this matter. We have added a brief discussion on this limitation in the Conclusion and Discussion section (refer to Page 16, Lines 22-23 and Page 17, Lines 1-4). For ease of reference, the related discussion is copied below.

On Page 16-17:

*In real data applications, we assessed the performance of spatial domain identification by measuring the consistency between the identified domains and the manual annotation provided by the pathologists. While we recognize that these manual annotations might not perfectly reflect the true segmentation of domains integrating both morphological and molecular information, using them as a benchmark remains a standard and widely accepted practice in spatial domain identification, as established by precedent in foundational work [2-4]…*

**Comment 4**: *Regarding Response to Referee3-Comment7: We appreciate the authors noting that none of the methods performed well in separating the fat region from the fibrous tissue. However, our concerns were more broad. Fat (and perhaps also other tissue domains) and* *histologically distinct but molecular difficult to characterize and, as such, integration methods that rely on molecular information may not adequately capture such tissue domains. A discussion of why none of the methods performed well in separating the fat region from the fibrous tissue is warranted.*

**Response**: We are grateful to the referee for prompting further discussion to enhance our manuscript. Accordingly, we have enriched the related discussion in the second paragraph of the Conclusion and Discussion section, specifically addressing the limitations of spatial domain identification methods that predominantly utilize molecular profiles. The revised discussion can be found on Page 17, Lines 4-11. Below, we provide the updated text for the referee’s convenience.

On Page 17:

*…Moreover, iIMPACT, alongside other spatial domain identification methods primarily relying on molecular profiles, exhibits limited capability in characterizing regions that are histologically distinct but have similar or low-quality gene expression. For instance, for the human breast cancer dataset, none of the methods effectively distinguished the fat region (in blue) from the fibrous tissue (in red) as per the manual annotation. The constrained number of cells in fat tissue results in a limited amount of gene expression measured, leading to low-quality molecular profiles and consequently unsatisfactory performance in identification of those domains.*

**Comment 5**: *Regarding Response to Referee3-Comment8: We appreciate the author's clarification that "cell type abundance data derived from HD-Staining encompasses comprehensive morphological details..." While "latent spatial domain-specific relative abundances of cell types parametrized by iIMPACT offers [an] approach to define and characterize identified spatial domains." We believe this text should be included in the manuscript and not just as a response to reviewer.*

**Response**: We apologize for the oversight of not including this discussion in our first revision and have now addressed it in our second revision. Specifically, we discuss the innovative aspect of integrating image profiles to improve spatial domain identification performance and interpretability at the start of the Conclusion and Discussion section (see Pages 15, Lines 21-23 and Page 16, Lines 1-7). The relevant text is provided below for the referee’s convenience.

On Page 15-16:

*…Numerous methods for spatial domain identification necessitate a dimensionality reduction step applied to the molecular profile, which compromises the clarity and direct interpretability of the identified spatial domains. However, iIMPACT fully leverages cellular-level information from histology images to improve clustering performance and increase interpretability. The cell type abundance data derived from HD-Staining encompasses comprehensive morphological details, such as cell growth pattern, cell-cell interaction, and cell interaction with the surrounding microenvironment, thereby improving the performance. On the other side, the latent spatial domain-specific relative abundance of cell types parametrized by iIMPACT offers a straightforward and user-friendly approach to define and characterize the identified spatial domains…*

**Comment 6***: Regarding Referee3-Comment9: We found the author's clarification of the description of gene expression richness provided in the reviewer response to be helpful in our understanding of the concept. We believe this text should be included in the manuscript and not just as a response to reviewer.*

**Response**: We thank the referee for the suggestion. We have included the related text in the Results section (see Page 9, Lines 19-22) during our initial discussion of interactive zone analysis. For ease of reference, the newly added sentence is copied below.

On Page 9:

*…We calculated the gene expression richness at each spot within the tumor boundary, immune boundary, and interactive zone, defining it as the percentage of genes exhibiting non-zero read counts. Note that this measure was taken at the spot level rather than the single-cell level…*

**Comment 7**: *Regarding Response to Referee3-Comment12: We agree with the authors that there is a lack of experimentally validated ground truths for estimating false positives. However, we still find it is important to explicitly clarify the number of genes identified by iIMPACT. For example, a reader's interpretation of the result could be different if iIMPACT identified 10/1020 with 2 being known true positives or if it identify 1000/1020 with 2 being known as true positives. We found this information difficult to discern from the supplementary figures alone.*

**Response**: We express our gratitude to the referee for drawing attention to this matter. We have listed the number of genes identified by iIMPACT and other methods for the four analyzed datasets in the newly created Table S2 in Supplementary Materials (attached below). We have also referenced Table S2 in the Results section (see Page 10, Lines 12-13) during our initial discussion of spaDEGs downstream analysis. For ease of reference, the newly added sentence and Table S2 are copied below. We hope this addition addresses the referee’s concern.

On Page 10:

*…The number of genes identified by each method, along with their overlaps with the referenced gene set, is detailed in Table S2 in the supplementary materials…*

**References**

1.	Magurran AE: *Ecological diversity and its measurement.* Springer Science & Business Media; 2013.

2.	Li Z, Zhou X: **BASS: multi-scale and multi-sample analysis enables accurate cell type clustering and spatial domain detection in spatial transcriptomic studies.** *Genome biology* 2022, **23:**168.

3.	Zhao E, Stone MR, Ren X, Guenthoer J, Smythe KS, Pulliam T, Williams SR, Uytingco CR, Taylor SEB, Nghiem P, et al: **Spatial transcriptomics at subspot resolution with BayesSpace.** *Nat Biotechnol* 2021, **39:**1375-1384.

4.	Hu J, Li X, Coleman K, Schroeder A, Ma N, Irwin DJ, Lee EB, Shinohara RT, Li M: **SpaGCN: Integrating gene expression, spatial location and histology to identify spatial domains and spatially variable genes by graph convolutional network.** *Nat Methods* 2021, **18:**1342-1351.

**Third round of review**

**Reviewer #3**

The authors have done a thorough job addressing our concerns. In particular, we find the direct comparison between iIMPACT on just the molecular profile versus image profile versus integrated approach versus manual annotation as presented in S15-S17 to be a valuable comparison. These new additions demonstrate how the incorporation of histological information in defining spatial domains in conjunction with transcriptional information provides orthogonal information and represents an important contribution independent of any methodological overlaps with existing methods.
